# Enhanced 2D Spiral Cine DENSE MRI Using Low‐Rank Denoising for Improved Apparent Signal‐to‐Noise Ratio, Spatial Resolution, Efficiency, Accuracy, and Accessibility

**DOI:** 10.1002/mrm.70331

**Published:** 2026-03-09

**Authors:** Shu‐Fu Shih, Yuxiao Wu, Siyue Li, Zhengyang Ming, Arutyun Pogosyan, Fei Han, Holden H. Wu, J. Paul Finn, Kim‐Lien Nguyen, Xiaodong Zhong

**Affiliations:** ^1^ Department of Radiological Sciences University of California, Los Angeles Los Angeles California USA; ^2^ Department of Bioengineering University of California, Los Angeles Los Angeles California USA; ^3^ Physics and Biology in Medicine Graduate Program University of California, Los Angeles Los Angeles California USA; ^4^ Division of Cardiology University of California, Los Angeles Los Angeles California USA; ^5^ Division of Cardiology VA Greater Los Angeles Healthcare System Los Angeles California USA; ^6^ MR R&D Collaborations, Siemens Medical Solutions USA, Inc Los Angeles California USA

**Keywords:** cardiovascular magnetic resonance, denoising, DENSE, signal‐to‐noise ratio, spiral MRI, strain imaging

## Abstract

**Purpose:**

To develop a denoising technique for displacement encoding with stimulated echoes (DENSE) MRI that improves spatial resolution, efficiency, and accuracy, and enhances accessibility by implementing DENSE MRI at 0.55 T.

**Methods:**

We developed a low‐rank denoising technique, which leverages multidimensional spiral cine DENSE MRI data for empirical noise estimation via Monte Carlo simulation combined with automatic noise suppression. Thirty‐six subjects (16 healthy, 20 with heart disease) were scanned at 3 T with breath‐hold standard‐resolution 2D cine DENSE (2.8 × 2.8 mm^2^) in a short‐axis slice of the heart. In 10 healthy subjects, high‐resolution DENSE with 1.2 × 1.2 mm^2^ was acquired. Apparent signal‐to‐noise ratio (SNR), phase SNR, scan efficiency (SNR per heartbeat per unit voxel size), and standard deviation of segmental circumferential myocardial strain (E_cc_) were compared with Wilcoxon signed‐rank tests (*p* < 0.05 considered significant). High‐resolution and standard‐resolution DENSE results were compared using Bland–Altman analysis. Lastly, we scanned seven healthy subjects at 0.55 T and 3 T, and compared E_cc_ results.

**Results:**

Apparent magnitude SNR, phase SNR and scan efficiency were significantly improved after denoising in both standard‐resolution and high‐resolution DENSE (all *p* < 0.01). Bland–Altman analysis showed denoised high‐resolution DENSE E_cc_ had smaller mean differences (non‐denoised: 0.028 vs. denoised: 0.009) and narrower limits of agreement (non‐denoised: [−0.072, 0.127] vs. denoised: [−0.048, 0.065]), indicating improved accuracy. Strain measurements from denoised DENSE at 0.55 T showed good agreement with those from 3 T, demonstrating feasibility of DENSE MRI at 0.55 T.

**Conclusion:**

Our proposed denoising technique may allow DENSE MRI with improved spatial resolution, efficiency, and accuracy, and enhanced accessibility at 0.55 T.

## 1 Introduction

Displacement encoding with stimulated echoes (DENSE) magnetic resonance imaging (MRI) is a valuable technique for myocardial strain evaluation [1]. This technique encodes tissue displacement into the phase of DENSE MRI data. Compared to the conventional tagging methods, DENSE MRI provides higher strain mapping resolution, more efficient, and user‐friendly postprocessing. Cine DENSE MRI with spiral acquisitions has been demonstrated to provide highly accurate [2, 3, 4, 5, 6] and reproducible [3, 7, 8, 9] strain measurements and is applied to study cardiac function in various patient cohorts. Despite the higher signal‐to‐noise ratio (SNR) efficiency of spiral trajectories [10] compared to other trajectories such as echo‐planar imaging (EPI) [11], the inherent SNR in DENSE MRI is still relatively low because stimulated echoes yield only half the SNR of conventional (non‐stimulated) echo acquisitions [12]. Low SNR can limit the spatial resolution, efficiency, and accuracy of strain measurements, depending on imaging protocols.

There has been growing interest in cardiac MRI at lower field strengths (< 1 T) because of several advantages, including reduced hardware costs, reduced siting requirements, and improved quality in imaging patients with implanted devices [13, 14, 15]. Lower field MRI can increase access to care with more cost‐effective systems. However, with the reduced equilibrium polarization at lower field strengths, the inherently low‐SNR DENSE MRI becomes more challenging. Techniques that can effectively improve SNR are crucial to enable DENSE MRI at lower field strengths.

Many denoising techniques have been developed for various MRI applications [16, 17, 18, 19], but their applications in cine DENSE MRI have not been well investigated. Previous works have performed smoothing on the displacement vectors to reduce the influence from low‐SNR images [20, 21] or investigated image filtering methods to improve strain measurements [22]. These previous attempts focused on improving displacement and strain results rather than the original DENSE images, and either required manual threshold selection or could not avoid signal over‐smoothing. These limit their applicability across various clinical applications and may impact the accuracy of strain measurement with high image resolutions.

In recent years, techniques for objective estimation of noise level in a low‐rank matrix model have become popular [18, 23, 24]. One promising approach is to use random matrix theory (RMT) denoising to improve image SNR, which leverages the low‐rank property in images and the characteristics of independent and identically distributed (i.i.d.) random Gaussian noise. This technique and its many variants have shown promising results in different MRI applications [18, 23, 24, 25]. Since spiral cine DENSE MRI involves data acquisition with multiple dimensions, including spatial, coil channel, cardiac phase, phase cycling, and displacement encoding dimensions, the redundancy in high‐dimensional DENSE data can be leveraged to generate patches for low‐rank denoising. However, the use of nonuniform fast Fourier Transform (NUFFT) involves convolution operations which create dependencies among neighboring gridded k‐space samples and RMT denoising cannot be directly applied. One technique proposed to “decorrelate” the gridded k‐space data to make noise characteristics satisfy the i.i.d. requirements [26]. Nevertheless, the size of the covariance matrix for spatial decorrelation will increase substantially as data dimension increases and the manipulation of k‐space contrast impacts the reconstructed images.

In this study, we investigated the noise characteristics and developed the first denoising technique, based on a low‐rank approach, to suppress noise in spiral cine DENSE MRI without removing tissue signal. We evaluated our proposed denoising pipeline using in vivo data with standard‐resolution and high‐resolution 2D spiral cine DENSE MRI. Quantitative analysis on apparent magnitude and phase SNR, scan efficiency, and accuracy of strain measurements was performed to evaluate the improvements achieved using the proposed denoising technique. We further demonstrated how the proposed denoising technique enabled spiral cine DENSE MRI on a lower‐field commercial 0.55 T scanner, previously not feasible, for improved accessibility. This was shown by comparing strain measurements at 0.55 T with those at 3 T.

## 2 Methods

### 2.1 Noise Characteristics in Images From Spiral MRI

Here we briefly describe the Marchenko–Pastur law [27]. Let us consider a 2D random matrix X with dimensions [p by q] (p≤q) whose entries are drawn from a Gaussian distribution of mean 0 and variance $\sigma^{2}$. The probability density function of the eigenvalues λ of the matrix $Y=\frac{1}{q}XX^{T}$, can be described as [27]:

$$p\left(\lambda |\sigma^{2},\gamma \right)=\left\{\begin{array}{l} \frac{\sqrt{\left(b-\lambda \right)\left(\lambda -a\right)}}{2\pi \gamma \lambda \sigma^{2}}\;\text{if}\;a\leq \lambda \leq b\\ 0\;\text{otherwise} \end{array}\right.$$

where $a=\sigma^{2}{\left(1-\sqrt{\gamma }\right)}^{2}$, $b=\sigma^{2}{\left(1+\sqrt{\gamma }\right)}^{2}$, γ=p/q. The lower bound a and upper bound b are related to the noise variance and the matrix aspect ratio γ. As detailed in previous works [18, 23, 24, 25], using this noise property, we can identify singular value components associated with Gaussian noise and suppress them with singular value thresholding or shrinkage.

In non‐Cartesian MRI reconstruction, the gridding process introduces dependencies in local k‐space, which was further transformed to dependencies in the image domain through inverse Fourier transform. A Gaussian matrix with correlations across the rows can be represented as $B=\Sigma^{1/2}X$, where Σ is the covariance matrix and X is a random matrix with i.i.d. Gaussian entries. The distribution of the eigenvalues of $\frac{1}{q}BB^{T}$ has been studied and is shown to be supported on a finite interval [28] (also see Figure S1), but the exact probability density function and its lower and upper bounds depend on the covariance matrix and the Gaussian noise variance. The lower bound $a_{B}$ and upper bound $b_{B}$ can be modeled as $a_{B}=\sigma^{2}L\left(\gamma ,\Sigma \right)$ and $b_{B}=\sigma^{2}U\left(\gamma ,\Sigma \right)$ where the functions L and U characterize how matrix aspect ratio γ and the covariance matrix Σ change $a_{B}$ and $b_{B}$. If there are no dependencies in the Gaussian matrix (i.e., Σ is an identity matrix), the lower bound and upper bound will be reduced to $a=\sigma^{2}{\left(1-\sqrt{\gamma }\right)}^{2}$ and $b=\sigma^{2}{\left(1+\sqrt{\gamma }\right)}^{2}$, as shown in the Marchenko–Pastur law. Given an aspect ratio γ and a covariance matrix Σ, the ratios $\frac{a}{a_{B}}=\frac{{\left(1-\sqrt{\gamma }\right)}^{2}}{L\left(\gamma ,\Sigma \right)}$, $\frac{b_{B}}{b}=\frac{U\left(\gamma ,\Sigma \right)}{{\left(1+\sqrt{\gamma }\right)}^{2}}$ and $\frac{b}{a}=\frac{\sigma^{2}{\left(1+\sqrt{\gamma }\right)}^{2}}{\sigma^{2}{\left(1-\sqrt{\gamma }\right)}^{2}}$ will be fixed. Therefore, the relationship between $b_{B}$ and $a_{B}$ can be characterized as $b_{B}=\left(\frac{b_{B}}{b}\right)\times \left(\frac{b}{a}\right)\times \left(\frac{a}{a_{B}}\right)\times a_{B}$.

The ratio $\frac{b}{a}$ can be calculated using the matrix aspect ratio γ, and $a_{B}$ can be estimated using the smallest eigenvalues value of the matrix $\frac{1}{q}BB^{T}$. The two ratios $\frac{b_{B}}{b}$ and $\frac{a}{a_{B}}$ are dependent on the covariance matrix, which can be difficult to calculate and varies for different spiral trajectories and data dimensions.

In this study, we proposed to use Monte Carlo simulation to estimate the covariance matrix and the two ratios $\frac{b_{B}}{b}$ and $\frac{a}{a_{B}}$. A noise‐only complex‐valued k‐space data with the same data dimensions as in vivo DENSE acquisitions were simulated. The data were gridded and transformed to the image domain. The two ratios $\frac{b_{B}}{b}$ and $\frac{a}{a_{B}}$ can be calculated for all the image patches. The mean value from 10 noise‐only simulated samples will be used as estimator for $\frac{b_{B}}{b}$ and $\frac{a}{a_{B}}$. In the actual denoising process, the upper bound of the eigenvalue associated with noise can be calculated $b_{B}=\left(\frac{b_{B}}{b}\right)\times \left(\frac{b}{a}\right)\times \left(\frac{a}{a_{B}}\right)\times a_{B}$. Eigenvalues smaller than the value of $b_{B}$ will be thresholded for noise suppression (see Figure S1).

### 2.2 DENSE Reconstruction Pipeline With Denoising

**FIGURE 1:**
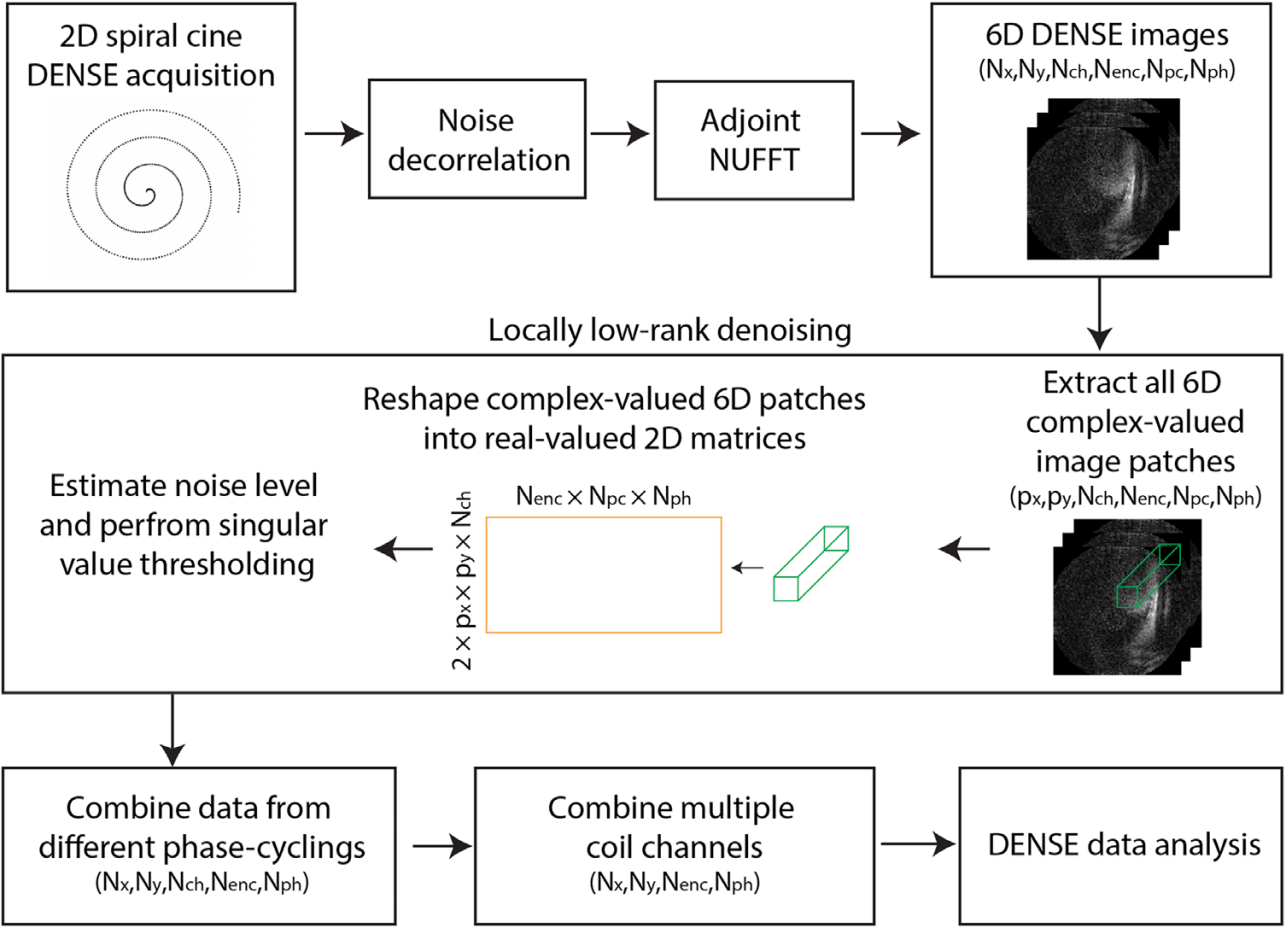
2D spiral cine DENSE MRI denoising and reconstruction pipeline.

The entire reconstruction pipeline is shown in Figure 1. First, the multicoil k‐space data of the 2D spiral cine DENSE data is decorrelated, so that the noise is pre‐whitened. After gradient delay correction and off‐resonance correction, adjoint NUFFT is performed on the spiral k‐space data to reconstruct 6D images with the size $\left[N_{x},N_{y},N_{\mathrm{ch}},N_{\mathrm{ph}},N_{\mathrm{pc}},N_{\mathrm{enc}}\right]$ where $N_{x}$ and $N_{y}$ represent the numbers of pixels in the two spatial dimensions of the DENSE image, $N_{\mathrm{ch}}$ represents the number of coil channels, $N_{\mathrm{ph}}$ represents the number of cardiac phases, $N_{\mathrm{pc}}$ represents the number of phase cycling, and $N_{\mathrm{enc}}$ represents the number of displacement encoding.

A patch size $p_{x}\times p_{y}$ needs to be selected prior to the denoising process. Local overlapping 6D image patches with dimensions $\left[p_{x},p_{y},N_{\mathrm{ch}},N_{\mathrm{ph}},N_{\mathrm{pc}},N_{\mathrm{enc}}\right]$ are extracted and reshaped into 2D matrices. This reshaping process requires selection of two sets of dimensions. One example is to group spatial x, spatial y and cardiac phase dimension into one set, and coil channel, phase cycling and displacement encoding dimensions into another. Additionally, MRI data are inherently complex‐valued. Therefore, the real and imaginary components need to be extracted and concatenated into one of the two sets. This results in a real‐valued 2D matrix whose rows and columns correspond to the respective combination with the size $2\times p_{x}\times p_{y}\times N_{\mathrm{ph}}$ by $N_{\mathrm{ch}}\times N_{\mathrm{pc}}\times N_{\mathrm{enc}}$. Previous studies often used the term Casorati matrix to describe a matrix with spatiotemporal or spatio‐parametric information [29], in which each column consists of image voxels from a subset of the data. In this work, the reshaped 2D matrix can have mixed spatial and the other dimension information (e.g., cardiac phase) in one column. Therefore, we will use the term Casorati‐like matrix to describe the reshaped 2D matrix in this work.

Singular value decomposition (SVD) is performed on the real‐valued 2D Casorati‐like matrix to obtain the singular values. The upper bound of singular values associated with noise was estimated using the method described in Section 2.1. The singular values smaller than the upper bound will be thresholded to suppress noise. After all the 2D Casorati‐like matrices are denoised, they are reshaped back to the original dimensions. The overlapping denoised patches are averaged to generate the final denoised 6D DENSE images. The denoised images from the phase cycled acquisitions are then combined. Images are further coil‐combined to generate the final DENSE magnitude and phase images for all the cardiac phases, followed by DENSE data analysis to yield the displacement and strain measurements.

All denoising steps are fully automated, given that the patch size and the dimension grouping strategy (for reshaping the 6D patches) are predetermined. Different patch sizes and different dimension grouping strategies were tested using numerical phantom data and in vivo data to evaluate their impact on denoising performance (in Section 2.3). Based on our experiments (results in Figures S2–S5), a 3 × 3 patch size with stride = 1 along both spatial x and spatial y was selected. The grouping combination (x,y,ch) by (ph,enc,pc) was selected, meaning that spatial x, spatial y and coil channel (“ch”) dimensions were in one set, and cardiac phase (“ph”), displacement encoding (“enc”), and phase cycling (“pc”) dimensions were in the other set.

The entire reconstruction pipeline, including the denoising step, was implemented in MATLAB (R2023a, MathWorks, Natick, MA) running on a 64‐Core 2.7 GHz CPU (AMD Ryzen Threadripper PRO 5995WX, Santa Clara, CA).

### 2.3 Numerical Phantom Experiment

We conducted a numerical phantom experiment to validate the denoising performance. We used the software tool DENSE‐SIM [30] to simulate “reference” numerical phantom data with realistic cardiac motion and no noise. The simulated reference 6D DENSE k‐space data had dimensions similar to the in vivo acquisitions (see the “high‐resolution DENSE protocol” in Section 2.4) with $N_{\mathrm{pts}}$ (number of spiral readout points) = 2784, N_sp_ (number of spiral interleaves) = 4, $N_{\mathrm{ch}}$ = 24, $N_{\mathrm{ph}}$ = 20, $N_{\mathrm{pc}}$ = 2, $N_{\mathrm{enc}}$ = 3. The image matrix size was set to $N_{x}$ = 128 and $N_{y}$ = 128. Additional sequence parameters, including displacement encoding frequency and through‐plane dephasing frequency, were all set to match with the high‐resolution in vivo scans acquired in this work.

We simulated low‐SNR DENSE data by adding complex‐valued Gaussian noise in the reference 6D k‐space data. We added noise such that the apparent SNR in the noisy magnitude images was similar to the in vivo high‐resolution (in‐plane 1.2 × 1.2 mm^2^) DENSE data. In this work, we used the definition of $\text{Apprent}\;\mathrm{SNR}=\frac{\mu }{\sigma }$, where μ represents the mean signal intensity in the (simulated) myocardium and σ represents the standard deviation of the background noise. We compared the choice of different dimension grouping and the choice of different patch sizes for denoising. To quantify the denoising performance, we calculated the normalized root mean squared error (NRMSE) between denoised images and the reference image.

### 2.4 Study Cohort and Data Acquisition—DENSE at 3 T

Our study was approved by the local institutional review board and was compliant with the Health Insurance Portability and Accountability Act.

A total of 36 subjects were recruited, with 16 healthy subjects (age 28 ± 8 years, female/male: 6/10, body mass index [BMI] = 25.2 ± 6.5 kg/m^2^) and 20 patients with heart disease (age 61 ± 16 years, female/male: 1/19, BMI = 30.5 ± 6.3 kg/m^2^). Among the 20 patients, nine had ischemic heart disease, and the other 11 were patients with various cardiovascular indications who underwent the routine clinical scans in our institutions. All subjects were scanned on clinical 3 T scanners (MAGNETOM Skyra or Vida, Siemens Healthineers, Forchheim, Germany). For each subject, a mid‐level short‐axis slice of the heart was acquired using a 2D spiral cine DENSE sequence [10, 31] with breath‐hold acquisitions. Imaging parameters included field‐of‐view (FOV) = 360 × 360 mm^2^, in‐plane resolution = 2.8 × 2.8 mm^2^, slice thickness = 8 mm, TR/TE = 16/1.08 ms, flip angle = 25°, cardiac phases = 16–28, temporal resolution = 25–44 ms (depending on heart rates), spiral interleaves per image = 6, spiral interleaves per heartbeat = 2, displacement encoding frequency = 0.10 cycles/mm, through‐plane dephasing frequency = 0.08 cycles/mm, acquisition time = 20 heartbeats.

Among the 16 healthy subjects, 10 of them were additionally scanned using a high‐resolution spiral cine DENSE protocol. Briefly, slice‐selective radio frequency (RF) pulses were used to excite a reduced FOV [32], thereby increasing the in‐plane resolution. Imaging parameters included FOV = 156 × 156 mm^2^, in‐plane resolution = 1.2 × 1.2 mm^2^, spiral interleaves per image = 4, acquisition time = 14 heartbeats, and other parameters the same as the standard‐resolution protocol. To improve readability, the terms “standard‐resolution DENSE” and “high‐resolution DENSE” are used throughout the remainder of this paper to refer to the datasets with the resolution 2.8 × 2.8 and 1.2 × 1.2 mm^2^, respectively. Results obtained at other image resolutions will be labeled with their corresponding values.

All cases were reconstructed with and without the proposed denoising technique. Patch size 3 × 3 and dimension grouping strategy (x,y,ch) by (ph,pc,enc) were used to denoise the images. This choice was based on our numerical phantom experiment results and image sharpness analysis on in vivo images (Figures S2–S5).

To study the denoising performance under various image resolutions in DENSE MRI, one of the healthy subjects was scanned with additional DENSE protocols with five different image resolutions: 2.8 × 2.8 mm^2^ (standard‐resolution), 2.0 × 2.0, 1.5 × 1.5, 1.2 × 1.2 mm^2^ (high‐resolution), 1.0 × 1.0 mm^2^, where other parameters were the same. DENSE acquisitions with these different spatial resolutions were scanned at the same slice location without subject repositioning.

### 2.5 Study Cohort and Data Acquisition—DENSE at 0.55 T, in Comparison to 3 T

Seven healthy subjects (age 30 ± 6 years, female/male: 2/5, BMI = 24.6 ± 4.0 kg/m^2^; cohort different from the subjects described in Section 2.4) were scanned on a commercially available 0.55 T scanner (MAGNETOM Free.Max, Siemens Healthineers, Forchheim, Germany). For each subject, a mid‐level short‐axis slice of the heart was acquired using a 2D breath‐hold spiral cine DENSE sequence [10] modified to adapt to the lower gradient performance. Sequence parameters were also adjusted because of the longer fat‐saturation pulse on the 0.55 T scanner. Detailed imaging parameters included FOV = 410 × 410 mm^2^, in‐plane resolution = 2.8 × 2.8 mm^2^, slice thickness = 8 mm, matrix size = 144 × 144, TR/TE = 24/1.49 ms, flip angle = 25°, cardiac phases = 15, temporal resolution = 50 ms, spiral interleaves per image = 6, spiral interleaves per heartbeat = 2, displacement encoding frequency = 0.10 cycles/mm, through‐plane dephasing frequency = 0.08 cycles/mm, acquisition time = 20 heartbeats. The images were reconstructed using the proposed denoising pipeline, with the same dimension grouping strategy and the same patch size used in the processing of 3 T DENSE data.

To validate the quantitative strain measurements at 0.55 T, the same subjects were also scanned at 3 T within a 2‐week interval. The sequence parameters for the scans at 3 T (MAGNETOM Prisma, Siemens Healthineers, Forchheim, Germany) were set as the same spatial and temporal resolutions as the 0.55 T data. Detailed imaging parameters included FOV = 360 × 360 mm^2^, in‐plane resolution = 2.8 × 2.8 mm^2^, slice thickness = 8 mm, matrix size = 128 × 128, TR/TE = 24/1.49 ms, flip angle = 25°, cardiac phases = 15, temporal resolution = 50 ms, spiral interleaves per image = 6, spiral interleaves per heartbeat = 2, displacement encoding frequency = 0.10 cycles/mm, through‐plane dephasing frequency = 0.08 cycles/mm, acquisition time = 20 heartbeats.

### 2.6 Quantitative Analysis on Apparent SNR and Scan Efficiency

We measured apparent magnitude and phase SNR and performed quantitative analysis to evaluate their improvement after denoising. The region of the left ventricular (LV) myocardium was manually annotated and used to calculate the mean signal intensity. A circular region of interest (ROI) was placed in the background region to measure the standard deviation of the noise with care to avoid image artifacts. The mean apparent magnitude SNR across all the cardiac phases was calculated for each subject. In addition, phase SNR was measured on the unwrapped phase images with a definition proposed in previous work [33]: $\text{phase}\;\mathrm{SNR}=\frac{\text{mean}\;\left(\text{phase of}\;\mathrm{mid}-\text{diastolic}\;\mathrm{ROI}\right)}{\mathrm{std}\;\left(\text{phase of}\;\mathrm{end}-\text{diastolic}\;\mathrm{ROI}\right)}$. To test whether apparent magnitude and phase SNR improvements after denoising were significant, we performed the Wilcoxon signed‐rank test with *p* < 0.05 considered significant. Statistical tests were conducted on both standard‐resolution and high‐resolution 3 T DENSE data.

We further calculated scan efficiency, which is defined as apparent magnitude SNR divided by acquisition time and voxel size. We compared scan efficiency for results from standard‐resolution and high‐resolution DENSE data with or without denoising. Scan efficiency comparison was performed in the 10 subjects who had both standard‐resolution and high‐resolution DESNE acquisitions, using the Wilcoxon signed‐rank test with *p* < 0.05 considered significant.

### 2.7 Quantitative Analysis on Image Sharpness

We conducted image sharpness analysis by measuring normalized image gradients. A one‐dimensional signal profile across the epicardial border was extracted from the magnitude image after manual annotation. From the signal profile, the normalized image gradient was defined as: $\frac{\left(I_{m}-I_{a}\right)}{I_{m}\times d}$, where $I_{m}$ is the signal intensity in the myocardium, $I_{a}$ is the signal intensity in the background, d is the difference between the two points. Wilcoxon signed‐rank statistical test is used to test whether the difference between with and without denoising is significant (if *p* < 0.05).

### 2.8 Quantitative Analysis on Strain Measurements for 3 T DENSE Data

After reconstruction, DENSE images were used to calculate displacement maps, E_cc_ strain maps and strain‐time curves in six mid‐level short‐axis LV segments using the workflow and algorithms outlined in previous work [10, 34, 35, 36]. Mean and standard deviation of segmental E_cc_ values across all the cardiac phases were recorded for both standard‐resolution and high‐resolution DENSE data. Because circumferential strain measured by MRI techniques including DENSE is generally associated with lower interobserver and interscan variability compared to other strain components [7, 37, 38, 39, 40, 41], this study focused on evaluating the circumferential strain before and after denoising, without compromising generalizability.

First, we performed Bland–Altman analysis to compare segmental E_cc_ values calculated from DENSE data before and after denoising. Mean difference (MD) and limits of agreement (LoA) were calculated. Second, we compared the standard deviations of segmental E_cc_ values before and after denoising. Wilcoxon signed‐rank statistical test is used to test whether the difference is significant (if *p* < 0.05).

### 2.9 Comparison of Strain Measurements in Different Image Resolutions at 3 T

We investigated the apparent SNR improvement and the agreement of segmental E_cc_ strain measurements in different image resolutions for DENSE at 3 T. In the first part, we compare apparent magnitude SNR and phase SNR among five different image resolutions (from 2.8 × 2.8 to 1.0 × 1.0 mm^2^) in one subject. In the second part, we investigated the agreement of strain measurements between standard‐resolution (2.8 × 2.8 mm^2^) and high‐resolution (1.2 × 1.2 mm^2^) DENSE. We performed Bland–Altman analysis to compare the segmental E_cc_ strain measurements in: (1) non‐denoised high‐resolution versus non‐denoised standard‐resolution, (2) denoised high‐resolution versus non‐denoised standard‐resolution, (3) non‐denoised high‐resolution versus denoised standard‐resolution, and (4) denoised high‐resolution versus denoised standard‐resolution. MD and LoA were used to investigate whether the proposed denoising technique would impact the accuracy of E_cc_ strain measurements using the standard‐resolution DENSE results as a reference standard. This analysis was done in the DENSE data of the 10 healthy subjects who had data acquired from two different resolutions and were the same subjects used for scan efficiency calculation in Section 2.6.

### 2.10 Comparison of Strain Measurements at 0.55 T to 3 T

We compared the strain measurement from denoised DENSE data at 0.55 T to 3 T in the seven subjects who went through scans at these two different field strengths. Bland–Altman analysis was performed by comparing the segmental E_cc_ measurements with MD and LoAs reported.

## 3 Results

### 3.1 Numerical Phantom Experiment Results

In our numerical phantom results, the proposed denoising techniques reduced the noise and achieved low NRMSE compared with non‐denoised images. Among the different dimension grouping strategies, we found (x, y, ch) by (ph, enc, pc) achieved the lowest NRMSE and more consistent phase information with the reference (Figure S2). Among the different patch sizes investigated (3 × 3 to 9 × 9), a small patch size of 3 × 3 performed better than larger patch sizes in terms of lower NRMSE (Figure S3).

### 3.2 Qualitative Results of Denoised DENSE Images and Strain Maps at 3 T

**FIGURE 2:**
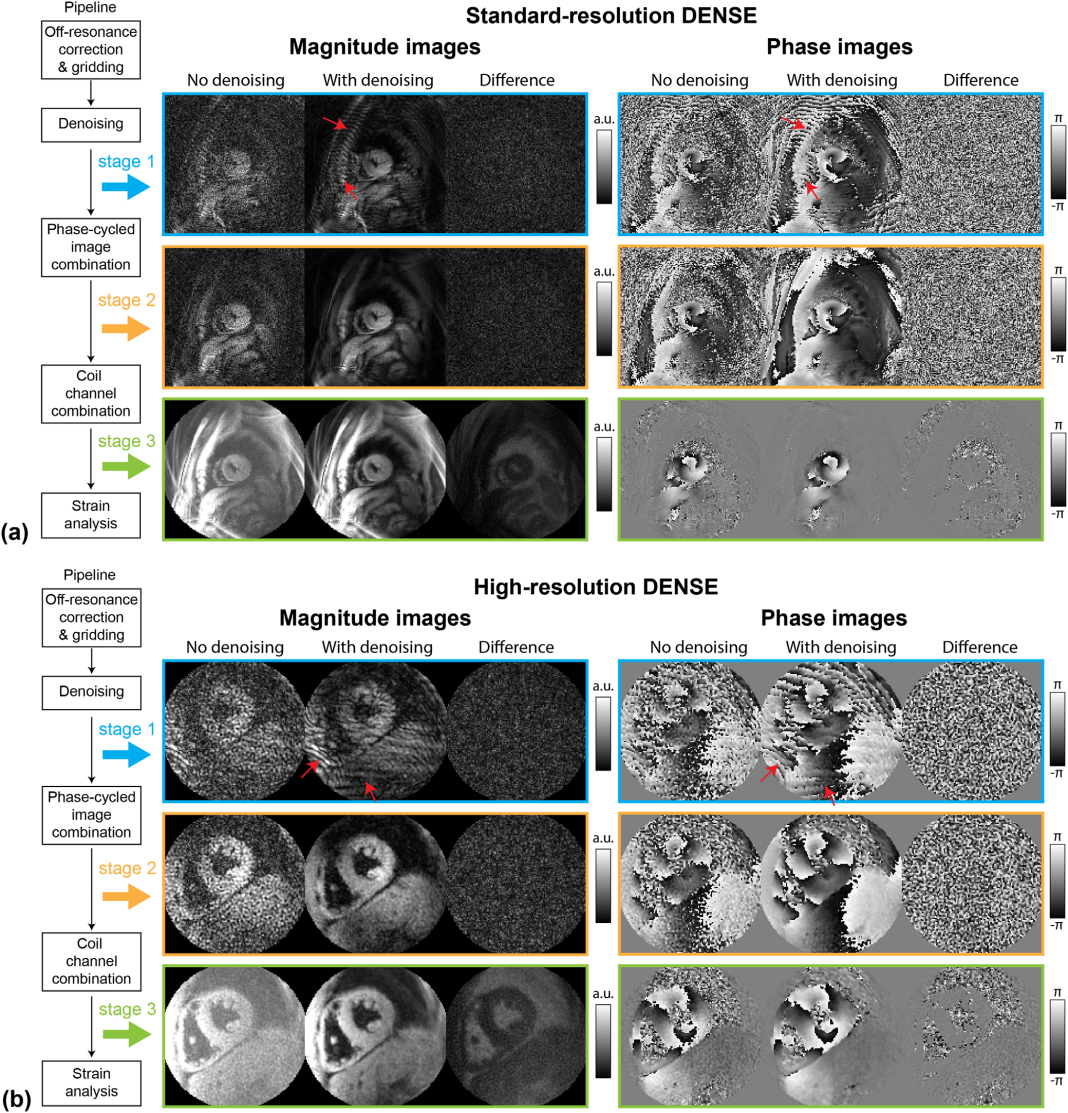
Representative denoising results at different reconstruction stages for (a) standard‐resolution in a patient with ischemic heart disease (female, 74‐year‐old) and (b) high‐resolution DENSE in a healthy volunteer (male, 34‐year‐old). Images were denoised using a patch size 3 × 3 and the dimension grouping strategy of ($p_{x}\times p_{y}\times N_{\mathrm{ch}}$) by ($N_{\mathrm{ph}}\times N_{\mathrm{pc}}\times N_{\mathrm{enc}}$). The proposed denoising technique only suppressed Gaussian noise, without removing structured signal (either tissue or the tagging artifacts, as indicated by red arrows). Window level settings were identical within each reconstruction stage and separately across different stages.

Representative denoising results in standard‐resolution and high‐resolution DENSE images from one patient and one healthy subject are shown in Figure 2. To demonstrate the robustness of the proposed denoising technique, we compared non‐denoised and denoised images at three different reconstruction stages: (1) before combination of phase‐cycled data, (2) before combination of multiple coil channels, and (3) the final reconstructed images. When comparing magnitude and phase images at the reconstruction stages before combination of phase‐cycled data and before combination of multiple coil channels, the signal difference did not show obvious structured signal and demonstrated the appearance of random noise. When comparing the magnitude and phase images at the final reconstruction stage, the noise in the background and in the myocardium was suppressed in both standard‐resolution and high‐resolution DENSE images.

**FIGURE 3:**
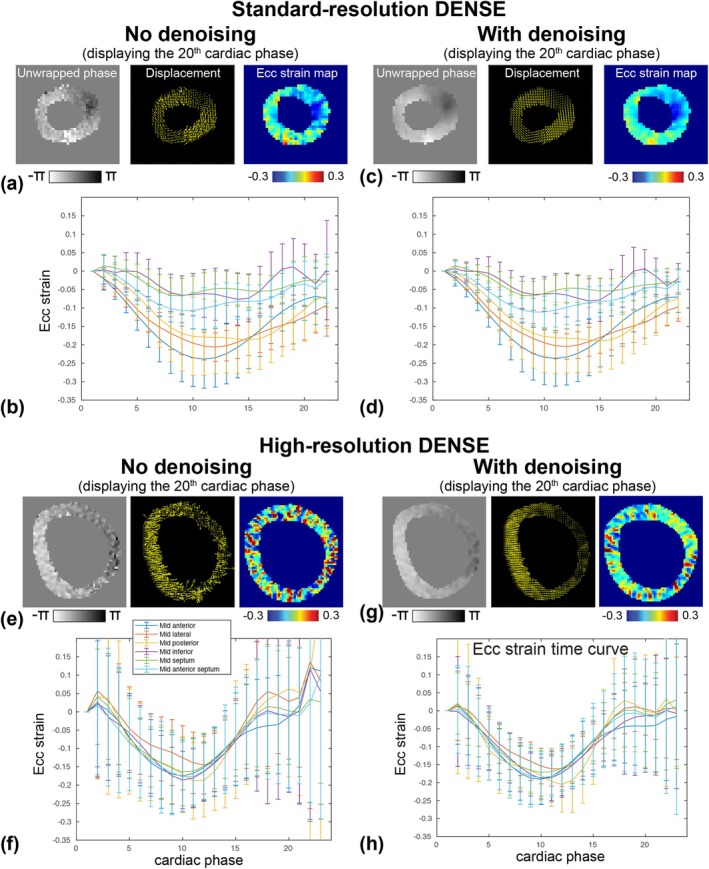
Comparison of unwrapped phase image, displacement map, E_cc_ strain map, and strain‐time curve for (a and b) non‐denoised standard‐resolution DENSE, (c and d) denoised standard‐resolution DENSE, (e and f) non‐denoised high‐resolution DENSE and (g and h) denoised high‐resolution DENSE. The standard‐resolution and high DENSE results were from the same patient and healthy subject as shown in Figure 2. Denoising reduced the noise in phase images (a vs. c and e vs. g). The E_cc_ strain‐time curves showed that the standard deviations of the strain measurements were reduced (b vs. d and f vs. h).

Representative denoising results of unwrapped phase images, displacement maps, E_cc_ strain maps, and E_cc_ strain‐time curves in standard‐resolution and high‐resolution DENSE from the same patient and healthy subject are shown in Figure 3. The unwrapped phase images have smoother phase transition in denoised DENSE results. This led to less noisy appearance in displacement maps and E_cc_ strain maps. In the standard‐resolution case, the E_cc_ strain‐time curves showed a reduction in standard deviations of segmental E_cc_ values during later cardiac phases, which typically have lower apparent SNR. In the high‐resolution case, erroneous fluctuations in the E_cc_ in the later cardiac phases (e.g., > 15) were corrected, and the standard deviation of segmental E_cc_ were largely reduced across all the cardiac phases, indicating improved accuracy.

### 3.3 Quantitative Results on Apparent Magnitude SNR, Phase SNR, and Scan Efficiency for DENSE at 3 T

**FIGURE 4:**
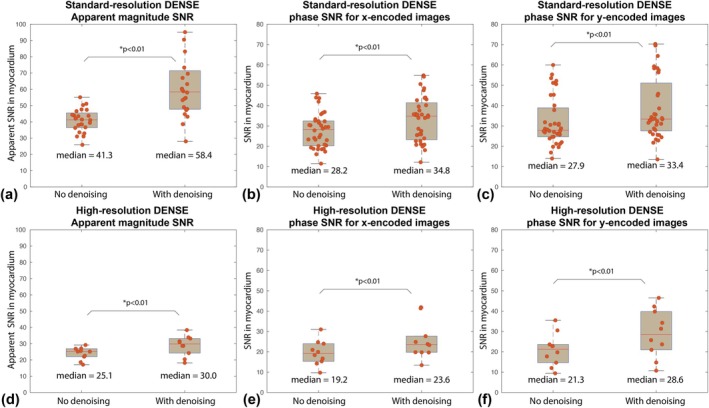
Box plots comparing apparent magnitude and phase SNR in the myocardium without and with denoising in standard‐resolution (a–c) and high‐resolution (d–f) DENSE MRI. Apparent magnitude SNR and phase SNR were significantly improved (all *p* < 0.01) after denoising in standard and high image resolutions.

The box plots comparing apparent magnitude SNR and phase SNR with and without denoising in standard‐resolution (*N* = 36) and high‐resolution (*N* = 10) DENSE are shown in Figure 4. For standard‐resolution DENSE data, the median of the apparent magnitude SNR was 41.3 and 58.4 in non‐denoised and denoised magnitude images. The phase SNR was improved from 28.2 to 34.8 in x‐encoded phase images and was improved from 27.9 to 33.4 in y‐encoded phase images. Wilcoxon statistical results showed that the apparent magnitude SNR and phase SNR were both significantly improved after denoising (all *p* < 0.01). For high‐resolution DENSE data, the median of the apparent magnitude SNR was 25.1 and 30.0 in non‐denoised and denoised magnitude images. The phase SNR was improved from 19.2 to 23.6 in x‐encoded phase images and was improved from 21.3 to 28.6 in y‐encoded phase images. Similar to the standard‐resolution DENSE, the apparent SNR improvements were all significant (all *p* < 0.01).

Scan efficiency was compared in the 10 subjects who had both standard‐resolution and high‐resolution DENSE acquisitions. Scan efficiency of non‐denoised and denoised was 0.0305 ± 0.004 and 0.0524 ± 0.025 (per heartbeat per mm^3^) for standard‐resolution DENSE at 3 T. Scan efficiency of nondenoised and denoised was 0.1445 ± 0.0225 and 0.1703 ± 0.0360 (per heartbeat per mm^3^) for high‐resolution DENSE at 3 T. Significant improvement in scan efficiency (both *p* < 0.01) were observed in both standard‐resolution and high‐resolution DENSE. A relative increase in scan efficiency after denoising was 70% for standard‐resolution DENSE and 18% for high‐resolution DENSE. The scan efficiency was around 3.2‐fold for denoised high‐resolution DENSE compared to denoised standard‐resolution DENSE.

### 3.4 Quantitative Results on Image Sharpness

In standard‐resolution datasets, normalized image gradients were 0.0160 ± 0.0388 in non‐denoised images and was significantly improved to 0.2428 ± 0.0201 after denoising (*p* < 0.01). In high‐resolution datasets, normalized image gradients were 0.0574 ± 0.0307 in non‐denoised images and was significantly improved to 0.1338 ± 0.0236 after denoising (*p* < 0.01). These results indicated improved image sharpness after denoising. More information regarding image sharpness analysis with different choices of grouping dimensions and patch sizes can be found in the Tables S1 and S2.

### 3.5 Quantitative Results on Strain Values for DENSE at 3 T

**FIGURE 5:**
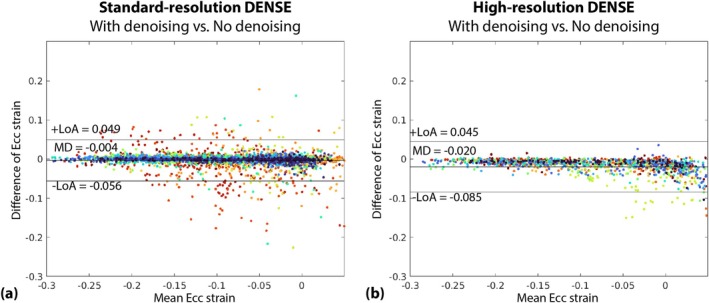
Bland–Altman plots comparing E_cc_ strain values with and without denoising in (a) standard‐resolution and (b) high‐resolution DENSE. Each dot represents one segmental E_cc_ strain measurements at one cardiac phase. All the E_cc_ strain measurements from the same subject were plotted with the same color.

Bland–Altman plots comparing the mean segmental E_cc_ values between non‐denoised and denoised DENSE are shown in Figure 5. MDs of E_cc_ between non‐denoised and denoised were −0.004 in standard‐resolution DENSE and −0.020 in high‐resolution DENSE; LoAs of E_cc_ between non‐denoised and denoised were [−0.056, 0.049] in standard‐resolution DENSE and [−0.085, 0.045] in high‐resolution DENSE. Good agreements of E_cc_ between non‐denoised and denoised DENSE were observed, while the LoA was slightly wider in high‐resolution DENSE compared with standard‐resolution DENSE. In addition, the Bland–Altman plot for high‐resolution DENSE showed an asymmetric distribution of data points, indicating systematic deviation between the denoised and non‐denoised results and highlighting the need for denoising in high‐resolution DENSE data.

**FIGURE 6:**
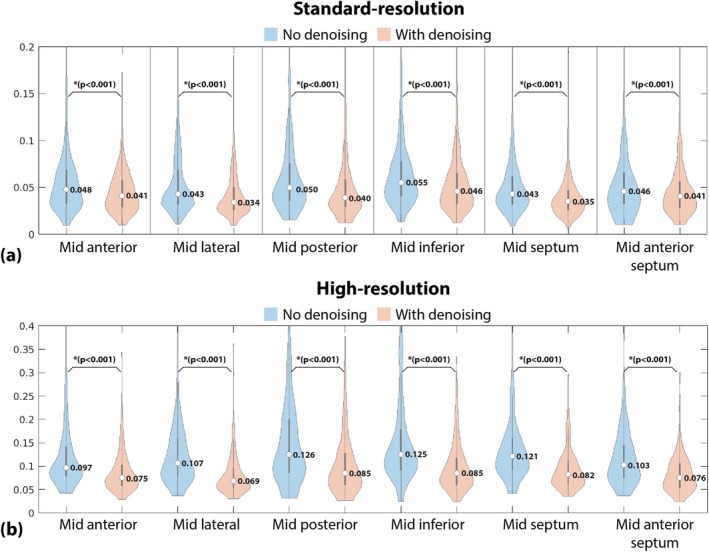
Violin plots comparing the standard deviation of E_cc_ strain measurements in six segments with and without denoising in (a) standard‐resolution and (b) high‐resolution DENSE. Denoising significantly reduced the standard deviations in both standard‐resolution and high‐resolution datasets (*p* < 0.001).

The violin plots comparing the standard deviations of segmental E_cc_ values between denoised and non‐denoised DENSE are shown in Figure 6. The standard deviations of segmental E_cc_ values were significantly (all with *p* < 0.001) reduced in all six segments of the mid‐level slice for both standard‐resolution and high‐resolution DENSE.

### 3.6 Comparison of 3 T DENSE With Different Spatial Resolutions

**FIGURE 7:**
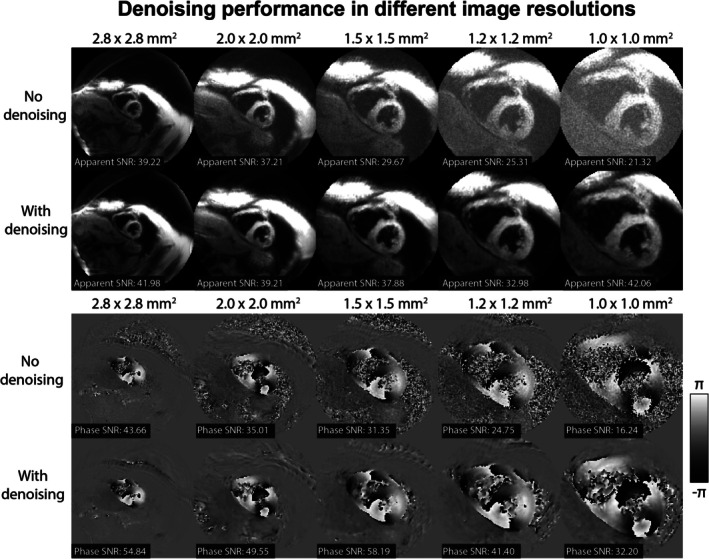
Comparison of DENSE image quality in different image resolutions. Apparent magnitude SNR and phase SNR at different image resolutions were measured and reported. Magnitude and phase images from all cardiac phases can be found in Videos S1 and S2.

Non‐denoised and denoised DENSE images at five different spatial resolutions in one subject are shown in Figure 7. Denoising improved both image apparent magnitude SNR and phase SNR at all different image resolutions.

**FIGURE 8:**
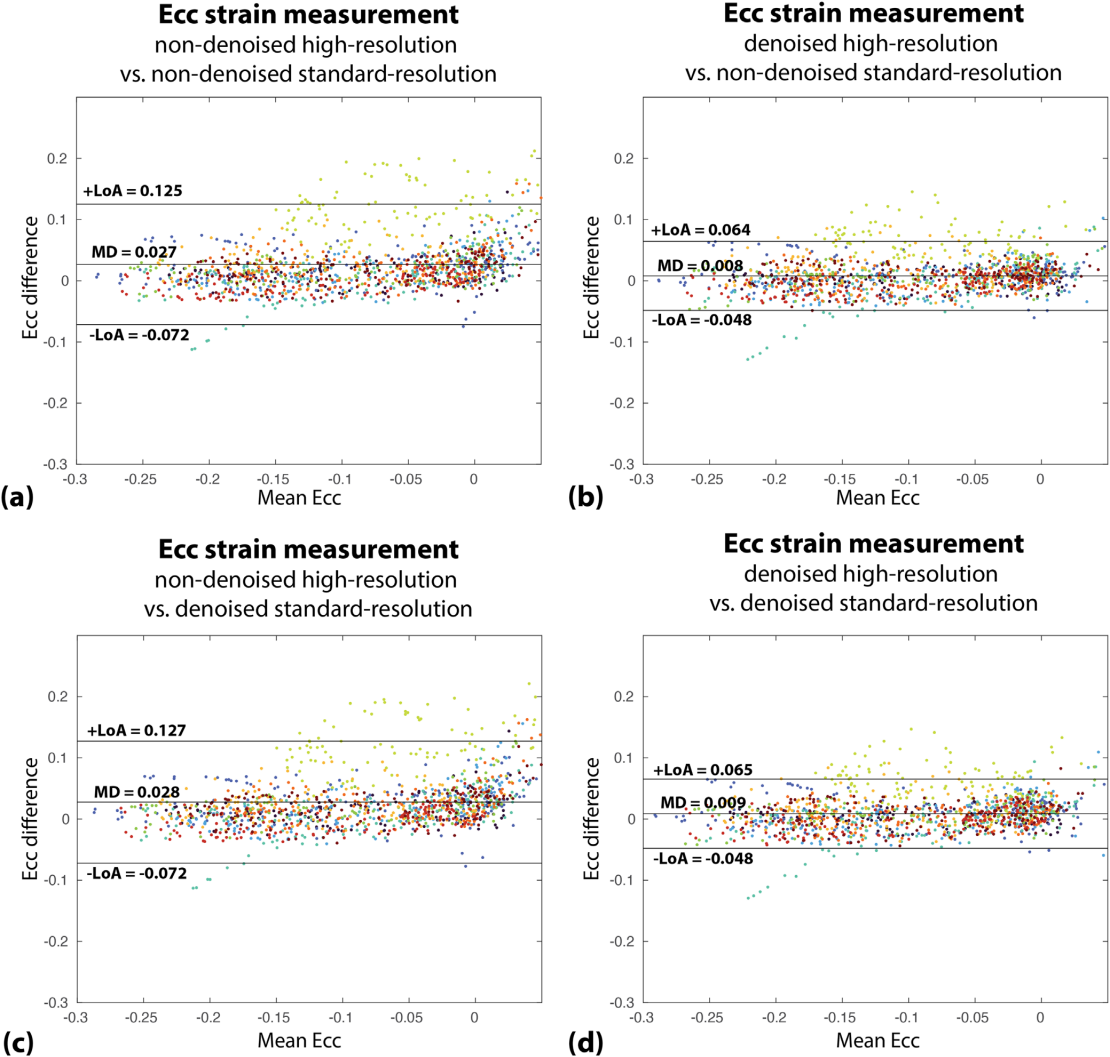
Bland–Altman plots comparing segmental E_cc_ strain measurements between high‐resolution and standard‐resolution DENSE. (a and b) Using non‐denoised standard‐resolution DENSE as the reference, denoised high‐resolution DENSE showed a smaller mean difference (MD) and narrower limits of agreement (LoA) compared to non‐denoised high‐resolution DENSE. (c and d) Using denoised standard‐resolution DENSE as the reference, denoised high‐resolution DENSE also showed a smaller MD and narrower LoA compared to non‐denoised high‐resolution DENSE. Each dot represents one segmental E_cc_ strain measurements at one cardiac phase. All the E_cc_ strain measurements from the same subject were plotted with the same color.

Bland–Altman analysis results comparing segmental E_cc_ strain measurements between two different image resolutions are shown in Figure 8. First, we used the segmental E_cc_ measurements of non‐denoised standard‐resolution DENSE as the reference standard (Figure 8a,b). The MD and LoA between non‐denoised high‐resolution and the reference were 0.027 and [−0.072, 0.125]; the MD and LoA between denoised high‐resolution and the reference were 0.008 and [−0.048, 0.064]. Denoised high‐resolution DENSE achieved better agreement with smaller MD and narrower LoA, reflecting improved accuracy for denoised high‐resolution DENSE data.

Second, we used the denoised standard‐resolution DENSE as the reference (Figure 8c,d). The MD and LoA between non‐denoised high‐resolution and the reference were 0.028 and [−0.072, 0.127]; the MD and LoA between denoised high‐resolution and the reference were 0.009 and [−0.048, 0.065]. This pair of comparisons also showed that denoised high‐resolution DENSE achieved better agreement with the reference, consistently demonstrating improved accuracy for denoised high‐resolution DENSE data.

### 3.7 Results of Denoised DENSE Images and Strain Maps at 0.55 T

**FIGURE 9:**
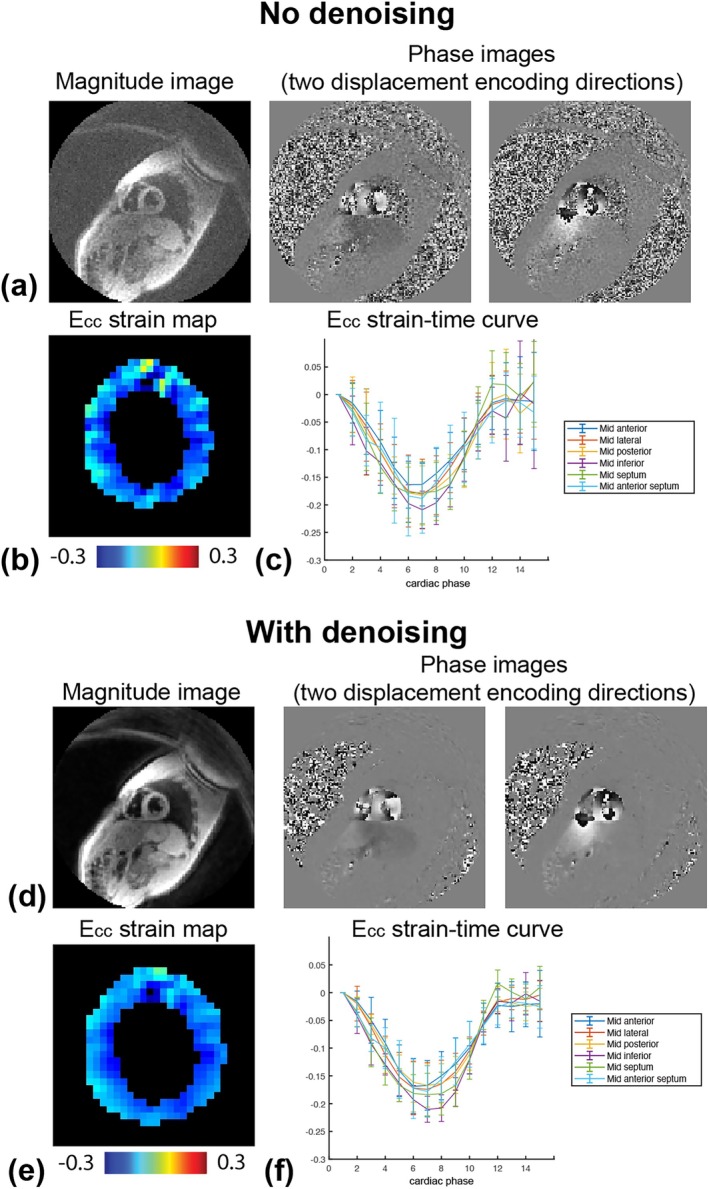
Representative of DENSE results at 0.55 T on the (a and d) magnitude image, phase image, (b and e) E_cc_ strain map, (c and f) segmental E_cc_ strain‐time curve without and with denoising in a 34‐year‐old male healthy subject. Noise was suppressed on magnitude and phase images. The standard deviations of segmental E_cc_ measurements were reduced after denoising. E_cc_ erroneous fluctuations were reduced in later cardiac phases after denoising. Images from all cardiac phases can be found in Video S3.

Representative non‐denoised and denoised DENSE magnitude images, phase images, E_cc_ strain map and E_cc_ strain‐time curves at 0.55 T from two healthy subjects are shown in Figures 9 and S6, respectively. Noise led to fluctuated strain measurements across the cardiac phases in results without denoising, especially in the late cardiac phases, and denoising effectively suppressed noise in both magnitude and phase images, resulting in more reliable strain‐time curves. Additionally, for both subjects, the standard deviations of E_cc_ values were reduced for all cardiac phases after denoising.

**FIGURE 10:**
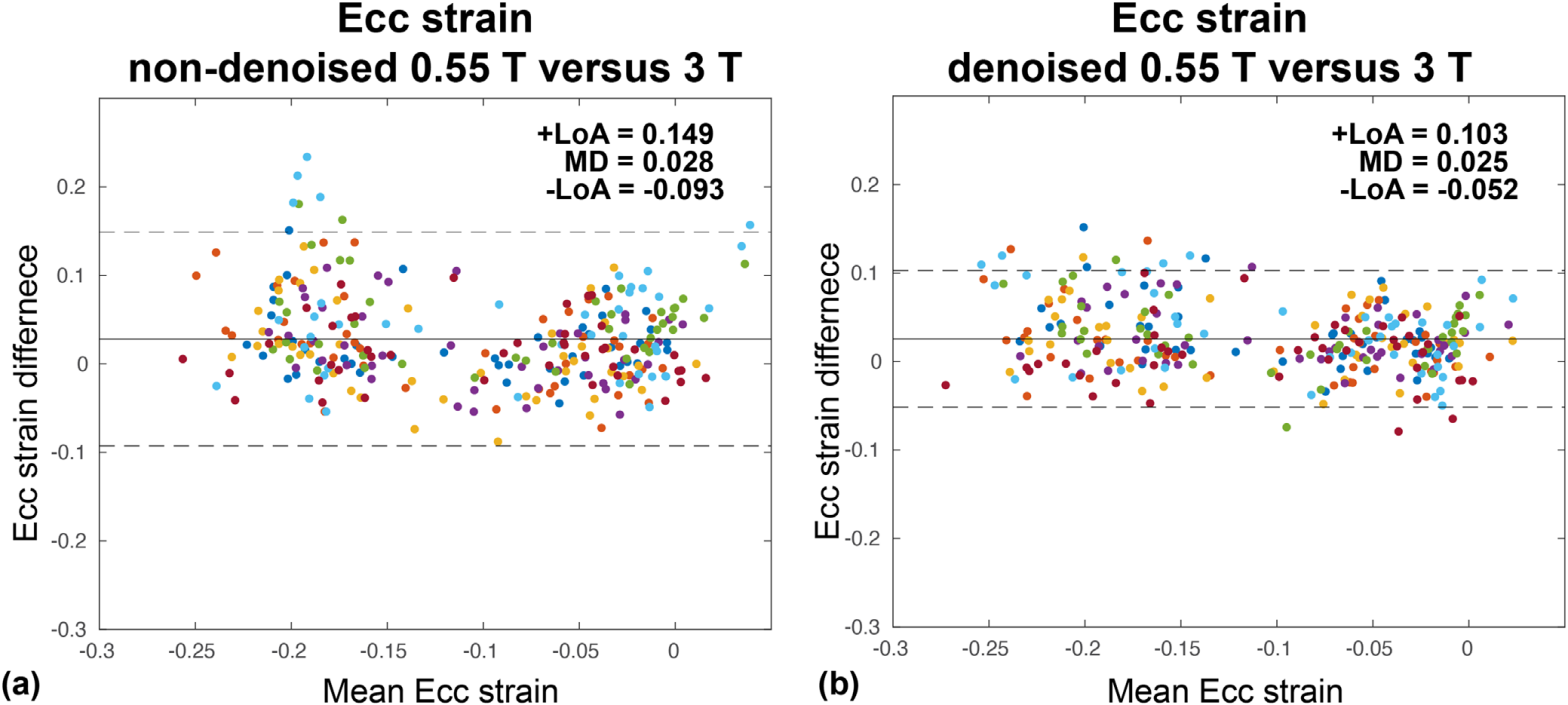
Bland–Altman plots of E_cc_ measurement in 7 healthy volunteers (a) between non‐denoised 0.55 T versus 3 T and (b) between denoised 0.55 T versus 3 T. All the E_cc_ strain measurements from the same subject were plotted with the same color.

Bland–Altman plots comparing E_cc_ strain in seven healthy volunteers between (1) non‐denoised 0.55 T versus 3 T and (2) denoised 0.55 T versus 3 T are shown in Figure 10a,b, respectively. Using 3 T results as the reference strain values, MD was 0.028 and 0.025 for non‐denoised and denoised 0.55 T, and LoAs were [−0.093, 0.149] and [−0.052, 0.103] for non‐denoised and denoised 0.55 T. After denoising, the LoA became narrower, showing improved agreement with the reference strain measurements.

### 3.8 Processing Time for Locally Low‐Rank Denoising

The processing time for the denoising step depends on the data dimension, including the number of coil channels and cardiac phases. For a representative standard‐resolution case (with 128 × 128 image matrix size, 24 coil channels, 22 cardiac phases, two phase cyclings, three DENSE encodings), the denoising step took 52 s. The corresponding processing time for the entire reconstruction pipeline, starting from reading raw k‐space data to saving image results, took 1 min 6 s.

Monte Carlo simulation of 10 instances (same data dimension as in the above paragraph) required around 8 min 55 s. The simulation process was done beforehand with many different combinations of parameters, with results saved (singular value thresholds) into a look‐up table. Therefore, the simulation time is reported separately and not included in the entire reconstruction time.

## 4 Discussion

In this study, we developed a low‐rank denoising technique to improve apparent SNR in spiral 2D cine DENSE MRI and evaluated it with in vivo data at different spatial resolutions and different field strengths. We assessed apparent magnitude and phase SNR, scan efficiency, and segmental E_cc_ strain measurements before and after denoising in healthy subjects and patients at 3 T, and in healthy subjects at 0.55 T. The standard deviation of segmental E_cc_ values during late cardiac phases (typically > 15) was reduced by denoising. The erroneous fluctuations in the strain‐time curves, specifically in 0.55 T DENSE and high‐resolution 3 T DENSE data, were corrected by denoising, demonstrating improved accuracy using the standard‐resolution 3 T DENSE data as a reference. Statistical analyses showed that apparent magnitude and phase SNR in both standard‐resolution and high‐resolution DENSE data were significantly improved with denoising. It was also revealed that segmental E_cc_ strain measurements exhibited better agreement between high‐resolution and standard‐resolution DENSE data with denoising, further showing improved accuracy. Significant improvements in scan efficiency were observed in both standard‐resolution and high‐resolution DENSE with denoising. This study is the first to implement DENSE MRI at 0.55 T. A modified spiral cine DENSE sequence with adjusted imaging parameters was developed to enable DENSE imaging at this field strength. In addition, we present the results at 0.55 T in conjunction with the proposed denoising technique. Together, these advances have the potential to improve accessibility to the DENSE MRI technique.

Denoising offers various advantages for DENSE image processing. First, a higher SNR enhances the delineation of the myocardium tissue boundaries, which helps achieve more robust segmentation for strain measurements. Second, reduced noise improves the stability of the phase unwrapping process, which is essential for accurate strain calculation. Third, denoising reduces the standard deviation in the strain results, which can potentially lower the measurement variability. Finally, higher SNR can be leveraged to achieve higher spatial resolution, faster acquisition times, or improved overall efficiency of DENSE MRI, as demonstrated in this study.

Even though the denoising pipeline can objectively estimate noise level, it is still necessary to select a proper patch size for constructing low‐rank matrices and a suitable dimension grouping strategy for better separation of signal and noise in the singular value spectrum. Our numerical phantom experiment showed that smaller patch sizes provide lower NRMSE compared with the reference, and the NRMSE slightly increased with the increased patch size. Our sharpness analysis in the in vivo data showed that denoising with larger patch sizes led to slightly smaller image gradients, indicating more blurring. On the other hand, the dimension grouping strategy had small impact on standard‐resolution DENSE but had different denoising performance in high‐resolution DENSE. We empirically found the dimension grouping strategy ($p_{x}\times p_{y}\times N_{\mathrm{ch}}$) by ($N_{\mathrm{ph}}\times N_{\mathrm{pc}}\times N_{\mathrm{enc}}$) and ($p_{x}\times p_{y}\times N_{\mathrm{ph}}$) by ($N_{\mathrm{ch}}\times N_{\mathrm{enc}}\times N_{\mathrm{pc}}$) worked generally well for standard‐resolution and high‐resolution in vivo DENSE data. An extended approach to select the patch size and the dimension grouping strategy in any given DENSE protocol setting including 4D DENSE data will be investigated in future studies.

Improving the spatial and temporal resolutions are two important needs for DENSE MRI in various clinical applications. The reduced FOV technique allows high spatial resolution DENSE MRI, with shorter acquisition time, as utilized in this study. A major limitation of high‐resolution DENSE is the reduction in SNR. In this study, we demonstrated that our proposed denoising technique enabled DENSE MRI acquisition with high resolution of 1.2 × 1.2 mm^2^ in better agreement with standard resolution DENSE compared to the same high‐resolution data without denoising. The results showed improved accuracy in high‐resolution DENSE data, establishing denoising as a critical step in the processing pipeline for such data. On the other hand, to improve temporal resolution, view sharing reconstruction has been employed in DENSE MRI, allowing additional cardiac phases to be calculated by incorporating data shared from neighboring phases. However, view sharing relies on temporally dependent information, which can alter noise characteristics, and is therefore not directly compatible with the proposed denoising technique. Nonetheless, with dedicated adjustments or adaptations, the proposed denoising technique holds promise for denoising view‐shared DENSE MRI data too, which warrants further investigation.

The computational time of the denoising step running on our workstation typically ranges from 45 s to 1 min 15 s depending on the data size. By implementing this method in C++ and running on higher‐performance hardware, this denoising method holds the potential to become faster and be integrated into the vendor reconstruction pipeline to perform inline reconstruction and display images soon after the acquisition.

Our study has limitations. First, we evaluated the proposed denoising technique only on short‐axis images, and the longitudinal and radial strain measurements were not investigated. We chose to validate our denoising technique in circumferential strain because it is more robust and used more often clinically. It has been known that radial strain is less reproducible than the circumferential strain because of the limited number of image pixels across the myocardium. The performance of the proposed denoising technique on longitudinal and radial strain measurements will be investigated in the future. Second, we achieved a fewer number of cardiac phases for DENSE at 0.55 T than 3 T due to the longer minimal TR at 0.55 T, if protocols were optimized independently at each individual field strength. The previously mentioned view sharing technique and further sequence development and optimization may partly mitigate this limitation. Third, we only evaluated a limited number of healthy subjects for DENSE at 0.55 T. However, patients with cardiovascular disease typically exhibit lower peak strain values and a narrower strain range compared with healthy subjects, and we compared strain values at all the time points in a cardiac cycle such that the investigated range of strain values covered the range that would be typically seen in patients. The overall quantitative performance of DENSE at 0.55 T in a larger cohort of subjects including patients with various cardiovascular diseases will require further research.

## 5 Conclusion

We proposed a low‐rank denoising technique to improve apparent SNR, scan efficiency and accuracy of strain measurements in 2D spiral cine DENSE MRI. At 3 T, denoised high‐resolution (1.2 × 1.2 mm^2^) DENSE provided more accurate and more precise strain measurements compared to non‐denoised high‐resolution DENSE when using standard‐resolution (2.8 × 2.8 mm^2^) DENSE as the reference. This study was the first to implement DENSE MRI on commercially available 0.55 T MRI, demonstrating the feasibility of DENSE MRI on low field with the proposed denoising technique. Our proposed denoising technique may allow DENSE MRI with improved spatial resolution, scan efficiency and accuracy, and enhanced accessibility at lower field strengths such as 0.55 T.

## Funding

This work was supported by the UCLA Academic Senate's Council on Research (COR), the Siemens Medical Solutions USA Inc, the Veterans Health Administration CSRD (I01‐CX001901), and the National Institutes of Health (R01HL148182).

## Conflicts of Interest

S.S. was previously partly supported by Siemens. F.H. is an employee of Siemens. X.Z. was a Siemens employee before this work was started. X.Z. received salary, stock, travel reimbursement, and other incentives from Siemens because of his Siemens employment, which were not directly associated with this study. X.Z. is currently an employee of the University of California, Los Angeles, and receives research funding and travel support from Siemens Medical Solutions USA Inc. X.Z. and Siemens have multiple full patents, provisional patents and invention disclosures, which are not directly associated with this study. The other authors declare no conflicts of interest.

## Supporting information


**Figure S1:** (a and b) Images reconstructed from simulated noise‐only k‐space data, (c, d, f, and g) signal intensity distributions at different image locations, and (e and h) the estimated probability density function of singular value distributions for spiral and Cartesian data through Monte Carlo simulation. The signal intensity distributions from spiral data are close to Gaussian and the normality test null hypothesis cannot be rejected (*p* = 0.652 and *p* = 0.745 for the two examples shown in the figure). Probability density function of singular value distribution in spiral data is different from that in Cartesian data due to data decencies from gridding. However, the probability density function is bounded and the ratio between upper bound of spiral noise data and theoretical value from Marcheko‐Pastur distribution stay fixed and can be estimated through Monte Carlo simulation.
**Figure S2:** Comparison of denoising performance in the simulated numerical phantom using different dimension grouping strategies. The different strategies all reduced the noise in the magnitude image and achieved a low normalized root mean squared error (NRMSE) compared to the reference image without noise. Slightly more phase difference was observed in results using dimension grouping strategies (x,y,enc) by (ch,ph,pc) and (x,y,pc) by (ch,ph,enc), as indicated by the red arrows. Among different dimension grouping strategies, (x,y,ch) by (ph,enc,pc) provided the smallest NRMSE and more consistent phase information with the reference.
**Figure S3:** Comparison of denoising performance in the simulated numerical phantom using different patch sizes for denoising. All different patch sizes reduced the noise and achieved a smaller normalized root mean squared error (NRMSE) compared to the reference image without noise. Consistent phase information with respect to the reference was also observed in results denoised with different patch sizes. Among different patch sizes, patch size 3 × 3 provided the smallest NRMSE.
**Figure S4:** Representative denoising results with dimension grouping strategies and the corresponding signal profiles for image sharpness analysis. The standard‐resolution and high‐resolution DENSE images were reconstructed and denoised using a patch size of 3 × 3 and four different dimension grouping strategies. Window level settings were identical within each row and separately across different rows. The red lines in (a) and (b) indicate the signal profile path used for image sharpness analysis.
**Figure S5:** Representative denoising results with different patch sizes and the corresponding signal profiles for image sharpness analysis. The standard‐resolution and high‐resolution DENSE images were reconstructed and denoised using the dimension grouping strategy ($p_{x}\times p_{y}\times N_{\mathrm{ch}}$) by ($N_{\text{phase}}\times N_{\mathrm{pc}}\times N_{\mathrm{enc}}$) with patch sizes ranging from 3 × 3 to 9 × 9. Window level settings were identical within each row and separately across different rows. The red lines in (a) and (b) indicate the signal profile path used for image sharpness analysis.
**Figure S6:** Representative of DENSE results at 0.55 T on the (a and d) magnitude image, phase image, (b and e) E_cc_ strain map, (c and f) segmental E_cc_ strain‐time curve without and with denoising from a 25‐year‐old female healthy subject. Noise was suppressed on magnitude and phase images. The standard deviations of segmental E_cc_ measurements were reduced after denoising. E_cc_ erroneous fluctuations were reduced in later cardiac phases after denoising. Images from all cardiac phases can be found in Video S4.
**Table S1:** Comparison of normalized image gradient in images denoised using different grouping dimensions. Mean and standard deviations of the image gradients from all the cardiac phases and subjects are reported. In standard‐resolution cases, different choices of dimension grouping strategies all provided similar sharpness in terms of normalized image gradients. In high‐resolution cases, denoising results from dimension grouping strategies of (x, y, ch) by (ph, enc, pc) and (x, y, ph) by (ch, enc, pc) provided higher normalized image gradients than the other two strategies. Based on this result, we chose the dimension grouping strategy of (x, y, ch) by (ph, enc, pc).
**Table S2:** Comparison of normalized image gradient in images denoised using different patch sizes. Mean and standard deviations of the image gradients from all the cardiac phases and subjects are reported. Denoised images using smaller patch sizes gave sharper images in terms of normalized image gradients. Same trends were observed in both standard‐resolution and high‐resolution data. Based on this result, we chose the patch size of 3 by 3 to perform denoising.

**Video S1:** Comparison of non‐denoised and denoised magnitude images at 3 T with image resolutions. Top row (from left to right): non‐denoised 2.8 × 2.8, 2.0 × 2.0, 1.5 × 1.5, 1.2 × 1.2, 1.0 × 1.0 mm2; bottom row (from left to right): denoised 2.8 × 2.8, 2.0 × 2.0, 1.5 × 1.5, 1.2 × 1.2, 1.0 × 1.0 mm^2^.

**Video S2:** Comparison of non‐denoised and denoised phase images at 3 T with different image resolutions. Top row (from left to right): non‐denoised 2.8 × 2.8, 2.0 × 2.0, 1.5 × 1.5, 1.2 × 1.2, 1.0 × 1.0 mm2; bottom row (from left to right): denoised 2.8 × 2.8, 2.0 × 2.0, 1.5 × 1.5, 1.2 × 1.2, 1.0 × 1.0 mm^2^.

**Video S3:** Comparison of non‐denoised and denoised cine DENSE images at 0.55 T (the same healthy subject as in Figure 9). Top row: non‐denoised magnitude image, and phase images with two displacement encoding directions; bottom row: denoised magnitude image, and phase images with two displacement encoding directions.

**Video S4:** Comparison of non‐denoised and denoised cine DENSE images at 0.55 T (the same subjects as in Figure S6). Top row: non‐denoised magnitude image, and phase images with two displacement encoding directions; bottom row: denoised magnitude image, and phase images with two displacement encoding directions.

## Data Availability

The data that support the findings of this study are available on request from the corresponding author. The data are not publicly available due to privacy or ethical restrictions.
