# Supplementary material for: Enhanced 2D Spiral Cine DENSE MRI Using Low‐Rank Denoising for Improved Apparent Signal‐to‐Noise Ratio, Spatial Resolution, Efficiency, Accuracy, and Accessibility
Source: Magn Reson Med. 2026 Mar 9;96(1):403–19. doi: 10.1002/mrm.70331 (PMC13050631; doi:10.1002/mrm.70331)
Supplement: Supplementary file 1 — Figure S1: (a and b) Images reconstructed from simulated noise‐only k‐space data, (c, d, f, and g) signal intensity distributions at different image locations, and (e and h) the estimated probability density function of singular value distributions for spiral and Cartesian data through Monte Carlo simulation. The signal intensity distributions from spiral data are close to Gaussian and the normality test null hypothesis cannot be rejected (p = 0.652 and p = 0.745 for the two examples shown in the figure). Probability density function of singular value distribution in spiral data is different from that in Cartesian data due to data decencies from gridding. However, the probability density function is bounded and the ratio between upper bound of spiral noise data and theoretical value from Marcheko‐Pastur distribution stay fixed and can be estimated through Monte Carlo simulation. Figure S2: Comparison of denoising performance in the simulated numerical phantom using different dimension grouping strategies. The different strategies all reduced the noise in the magnitude image and achieved a low normalized root mean squared error (NRMSE) compared to the reference image without noise. Slightly more phase difference was observed in results using dimension grouping strategies (x,y,enc) by (ch,ph,pc) and (x,y,pc) by (ch,ph,enc), as indicated by the red arrows. Among different dimension grouping strategies, (x,y,ch) by (ph,enc,pc) provided the smallest NRMSE and more consistent phase information with the reference. Figure S3: Comparison of denoising performance in the simulated numerical phantom using different patch sizes for denoising. All different patch sizes reduced the noise and achieved a smaller normalized root mean squared error (NRMSE) compared to the reference image without noise. Consistent phase information with respect to the reference was also observed in results denoised with different patch sizes. Among different patch sizes, patch size 3 × 3 provided the s [file MRM-96-403-s002.docx]

**Supplementary Information**


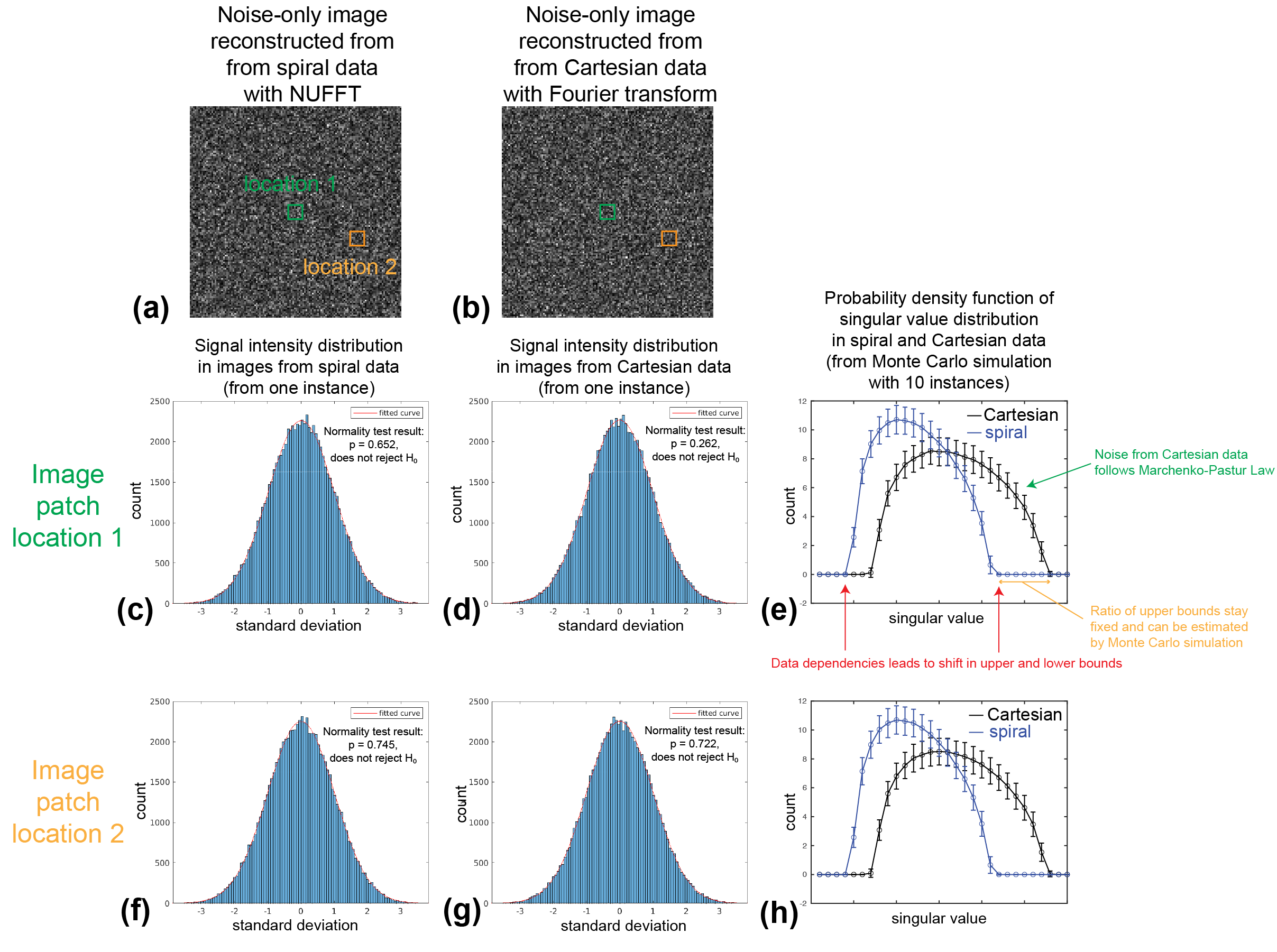


**Supplementary Figure S1**. **(a,b)** Images reconstructed from simulated noise-only k-space data, **(c,d,f,g)** signal intensity distributions at different image locations, and **(e,h)** the estimated probability density function of singular value distributions for spiral and Cartesian data through Monte Carlo simulation. The signal intensity distributions from spiral data are close to Gaussian and the normality test null hypothesis cannot be rejected (p=0.652 and p=0.745 for the two examples shown in the figure). Probability density function of singular value distribution in spiral data is different from that in Cartesian data due to data decencies from gridding. However, the probability density function is bounded and the ratio between upper bound of spiral noise data and theoretical value from Marcheko-Pastur distribution stay fixed and can be estimated through Monte Carlo simulation.

**
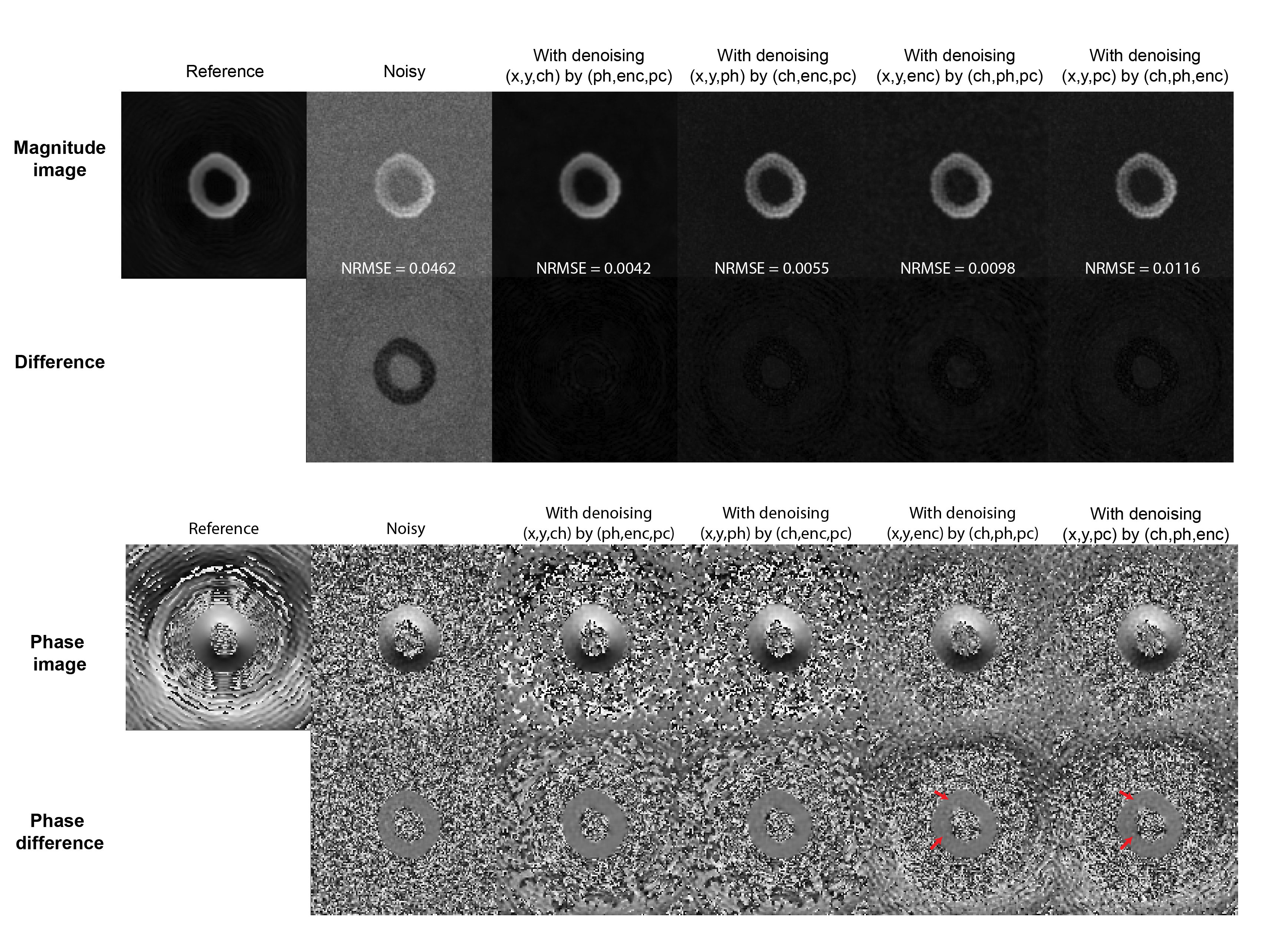
**

**Supplementary Figure S2.** Comparison of denoising performance in the simulated numerical phantom using different dimension grouping strategies. The different strategies all reduced the noise in the magnitude image and achieved a low normalized root mean squared error (NRMSE) compared to the reference image without noise. Slightly more phase difference was observed in results using dimension grouping strategies (x,y,enc) by (ch,ph,pc) and (x,y,pc) by (ch,ph,enc), as indicated by the red arrows. Among different dimension grouping strategies, (x,y,ch) by (ph,enc,pc) provided the smallest NRMSE and more consistent phase information with the reference.

**
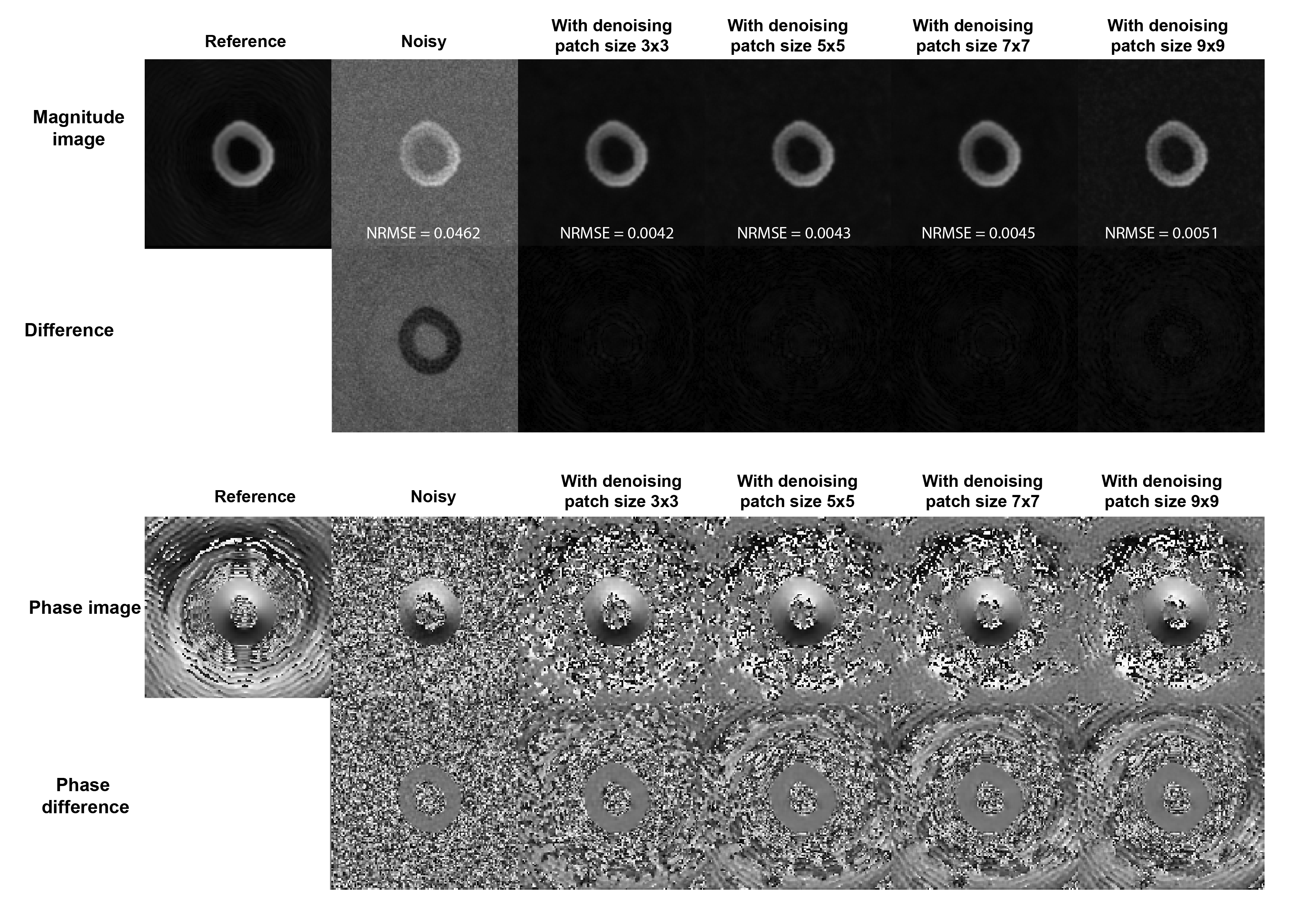
**

**Supplementary Figure S3.** Comparison of denoising performance in the simulated numerical phantom using different patch sizes for denoising. All different patch sizes reduced the noise and achieved a smaller normalized root mean squared error (NRMSE) compared to the reference image without noise. Consistent phase information with respect to the reference was also observed in results denoised with different patch sizes. Among different patch sizes, patch size 3$\times$3 provided the smallest NRMSE.

**
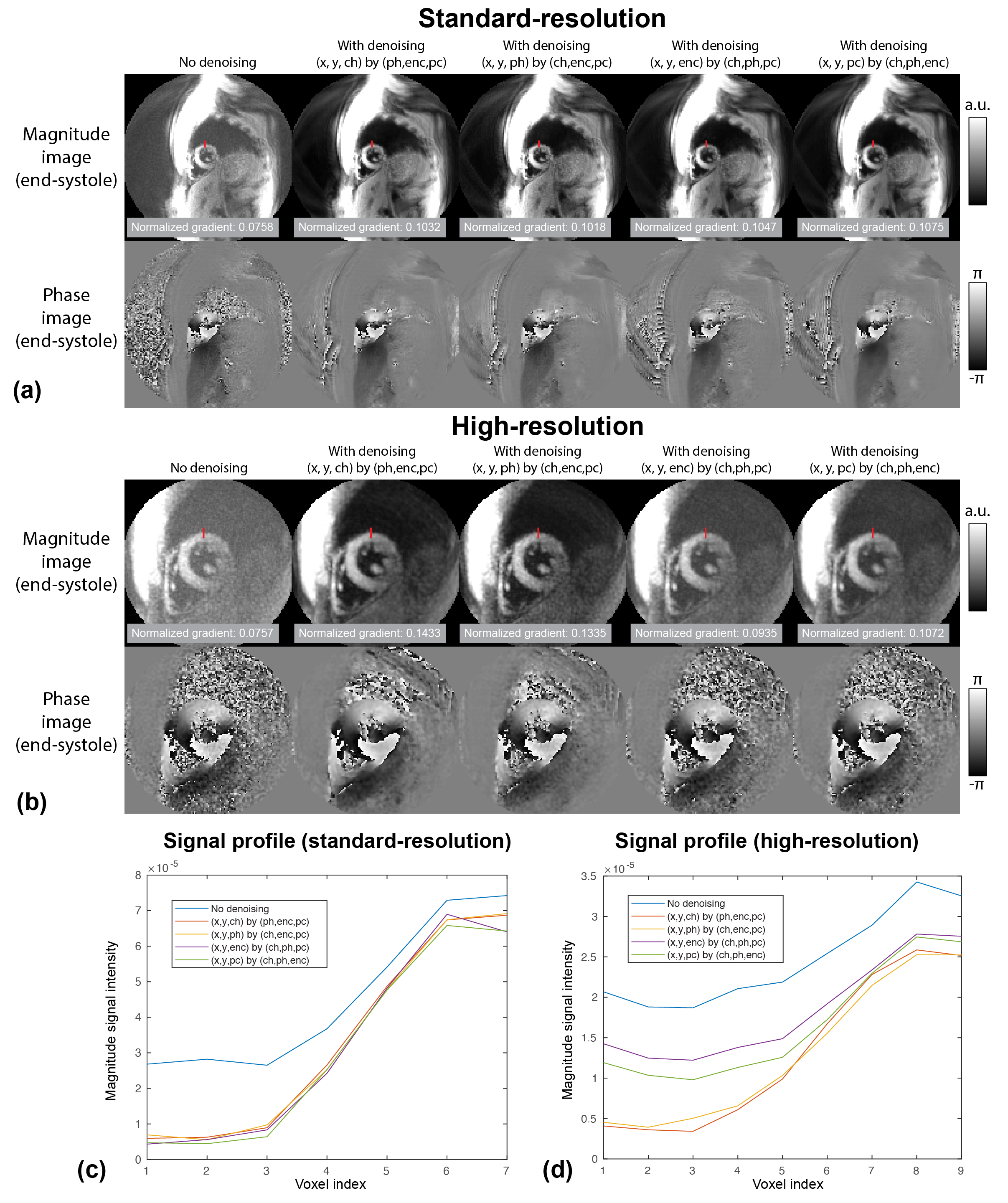
**

**Supplementary Figure S4**. Representative denoising results with dimension grouping strategies and the corresponding signal profiles for image sharpness analysis. The standard-resolution and high-resolution DENSE images were reconstructed and denoised using a patch size of 3$\times$3 and four different dimension grouping strategies. Window level settings were identical within each row and separately across different rows. The red lines in **(a)** and **(b)** indicate the signal profile path used for image sharpness analysis.

**
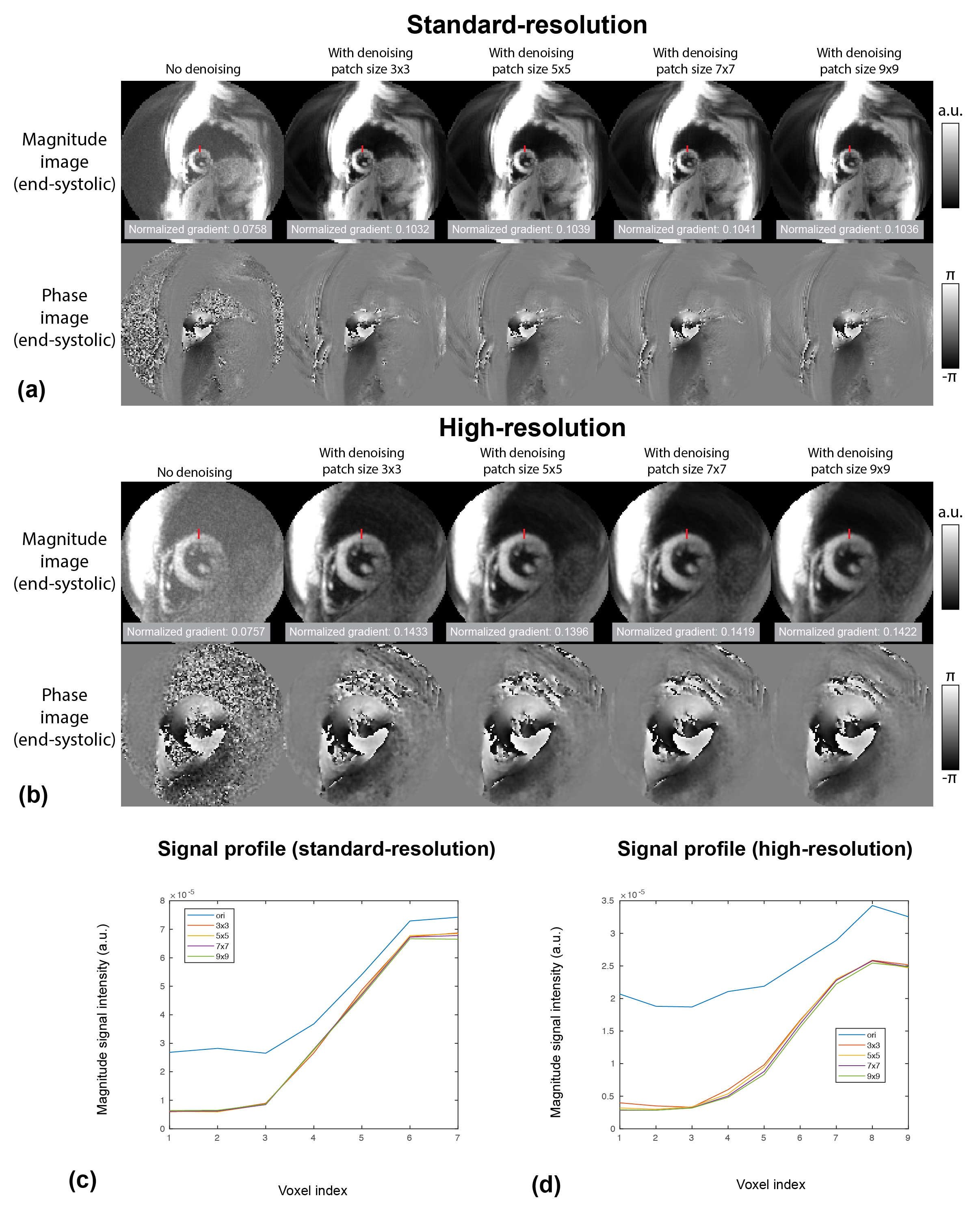
**

**Supplementary Figure S5**. Representative denoising results with different patch sizes and the corresponding signal profiles for image sharpness analysis. The standard-resolution and high-resolution DENSE images were reconstructed and denoised using the dimension grouping strategy ($p_{x}\times p_{y}\times N_{ch}$) by ($N_{phase}\times N_{pc}\times N_{enc}$) with patch sizes ranging from 3$\times$3 to 9$\times$9. Window level settings were identical within each row and separately across different rows. The red lines in **(a)** and **(b)** indicate the signal profile path used for image sharpness analysis.


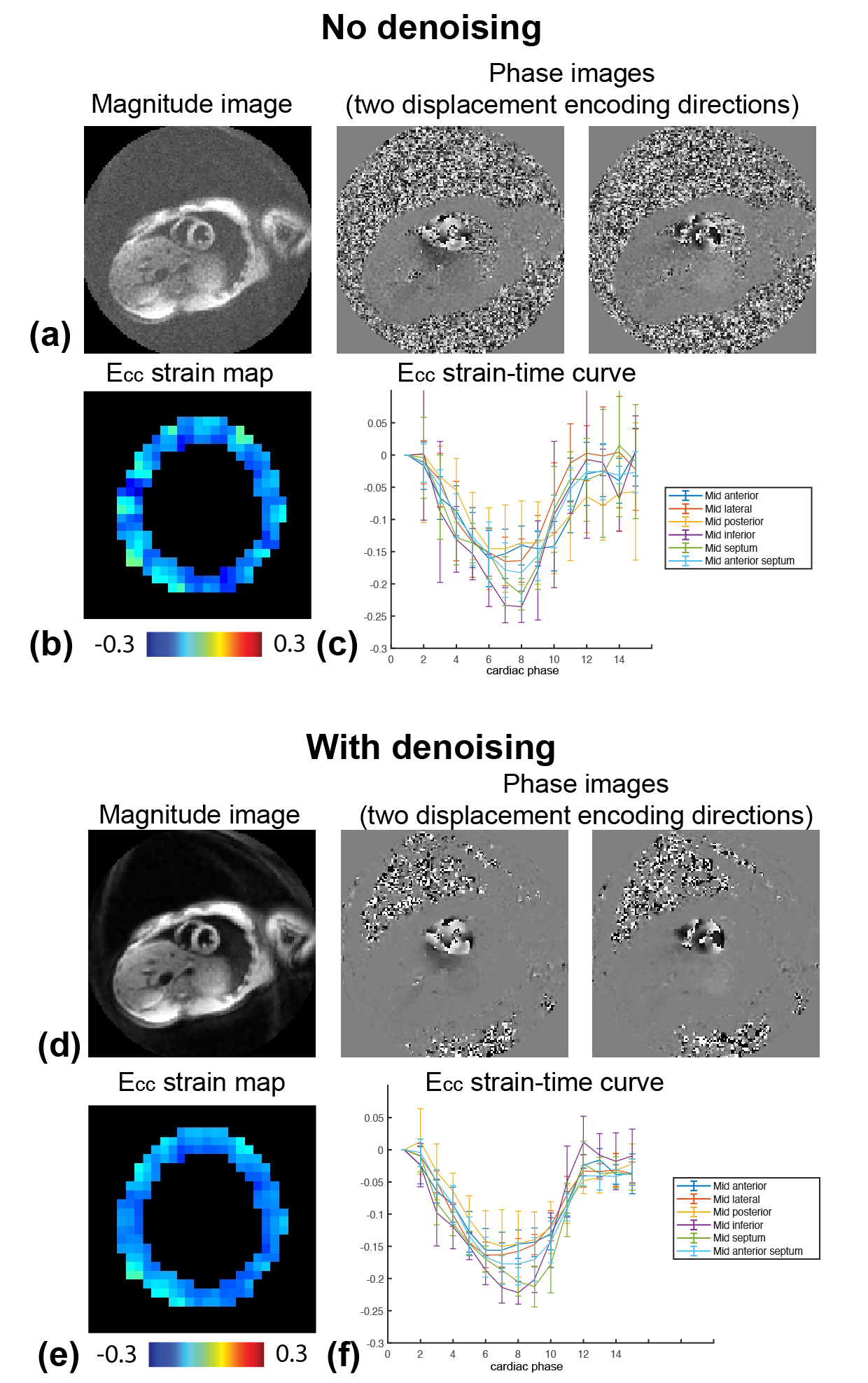


**Supplementary Figure S6**. Representative of DENSE results at 0.55 T on the **(a,d)** magnitude image, phase image, **(b,e)** E_cc_ strain map, **(c,f)** segmental E_cc_ strain-time curve without and with denoising from a 25-year-old female healthy subject. Noise was suppressed on magnitude and phase images. The standard deviations of segmental E_cc_ measurements were reduced after denoising. E_cc_ erroneous fluctuations were reduced in later cardiac phases after denoising. Images from all cardiac phases can be found in **Supplementary Video S4**.

**Supplementary Table S1**. Comparison of normalized image gradient in images denoised using different grouping dimensions. Mean and standard deviations of the image gradients from all the cardiac phases and subjects are reported. In standard-resolution cases, different choices of dimension grouping strategies all provided similar sharpness in terms of normalized image gradients. In high-resolution cases, denoising results from dimension grouping strategies of (x, y, ch) by (ph, enc, pc) and (x, y, ph) by (ch, enc, pc) provided higher normalized image gradients than the other two strategies. Based on this result, we chose the dimension grouping strategy of (x, y, ch) by (ph, enc, pc).

We also performed Wilcoxon signed-rank statistical test to compare the normalized image gradient results between non-denoised and denoised images. Denoising with four different dimension grouping strategies all showed significantly (all p<0.01) higher normalized image gradients when compared to non-denoised images. (** indicates p<0.01)

| Dimension grouping strategy | Standard-resolution | High-resolution |
| --- | --- | --- |
| No denoising | 0.0160$\pm$0.0388 | 0.0574$\pm$0.0307 |
| (x, y, ch) by (ph, enc, pc) | 0.2428$\pm$0.0201** | 0.1338$\pm$0.0236** |
| (x, y, ph) by (ch, enc, pc) | 0.2359$\pm$0.0226** | 0.1321$\pm$0.0238** |
| (x, y, enc) by (ch, ph, pc) | 0.2414$\pm$0.0192** | 0.0790$\pm$0.0218** |
| (x, y, pc) by (ch, ph, enc) | 0.2357$\pm$0.0213** | 0.0945$\pm$0.0292** |

**Supplementary Table S2**. Comparison of normalized image gradient in images denoised using different patch sizes. Mean and standard deviations of the image gradients from all the cardiac phases and subjects are reported. Denoised images using smaller patch sizes gave sharper images in terms of normalized image gradients. Same trends were observed in both standard-resolution and high-resolution data. Based on this result, we chose the patch size of 3 by 3 to perform denoising.

We also performed Wilcoxon signed-rank statistical test to compare the normalized image gradient results between non-denoised and denoised images. Denoising results with four different patch sizes all showed significantly (all p<0.01) higher normalized image gradients when compared to non-denoised images. (** indicates p<0.01)

| Patch size | Standard-resolution | High-resolution |
| --- | --- | --- |
| No denoising | 0.0160$\pm$0.0388 | 0.0574$\pm$0.0307 |
| 3 by 3 | 0.2428$\pm$0.0201** | 0.1338$\pm$0.0236** |
| 5 by 5 | 0.2427$\pm$0.0183** | 0.1320$\pm$0.0202** |
| 7 by 7 | 0.2414$\pm$0.0164** | 0.1311$\pm$0.0188** |
| 9 by 9 | 0.2394$\pm$0.0165** | 0.1303$\pm$0.0178** |


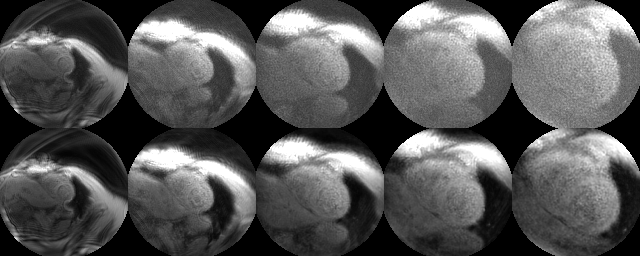


**Supplementary Video S1**. Comparison of non-denoised and denoised magnitude images at 3T with image resolutions. Top row (from left to right): non-denoised 2.8$\times$2.8 mm^2^, 2.0$\times$2.0 mm^2^, 1.5$\times$1.5 mm^2^, 1.2$\times$1.2 mm^2^, 1.0$\times$1.0 mm^2^; bottom row (from left to right): denoised 2.8$\times$2.8 mm^2^, 2.0$\times$2.0 mm^2^, 1.5$\times$1.5 mm^2^, 1.2$\times$1.2 mm^2^, 1.0$\times$1.0 mm^2^.


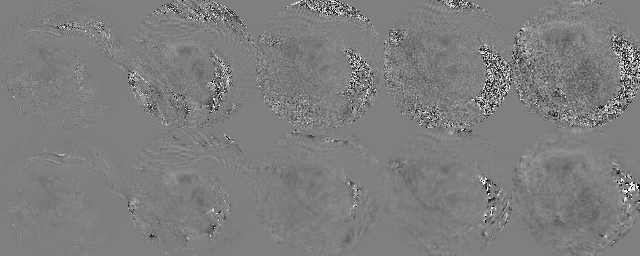


**Supplementary Video S2**. Comparison of non-denoised and denoised phase images at 3T with different image resolutions. Top row (from left to right): non-denoised 2.8$\times$2.8 mm^2^, 2.0$\times$2.0 mm^2^, 1.5$\times$1.5 mm^2^, 1.2$\times$1.2 mm^2^, 1.0$\times$1.0 mm^2^; bottom row (from left to right): denoised 2.8$\times$2.8 mm^2^, 2.0$\times$2.0 mm^2^, 1.5$\times$1.5 mm^2^, 1.2$\times$1.2 mm^2^, 1.0$\times$1.0 mm^2^.


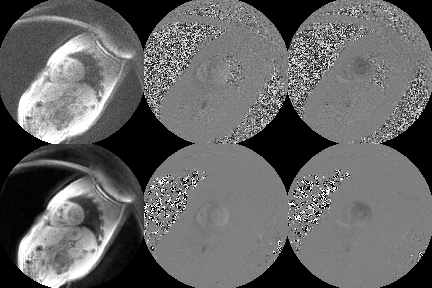


**Supplementary Video S3**. Comparison of non-denoised and denoised cine DENSE images at 0.55T (the same healthy subject as in **Figure 9**). Top row: non-denoised magnitude image, and phase images with two displacement encoding directions; bottom row: denoised magnitude image, and phase images with two displacement encoding directions.


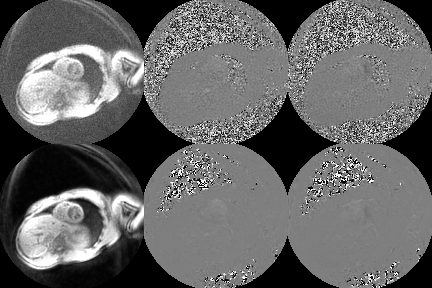


**Supplementary Video S4**. Comparison of non-denoised and denoised cine DENSE images at 0.55T (the same subjects as in **Supplementary Figure S6**). Top row: non-denoised magnitude image, and phase images with two displacement encoding directions; bottom row: denoised magnitude image, and phase images with two displacement encoding directions.
